# Discovery of a New Triterpenoid and Chemical Profiling of Mudjala (*Barringtonia acutangula*), an Australian Indigenous Medicinal Plant

**DOI:** 10.3390/molecules31193388

**Published:** 2026-09-23

**Authors:** Tabassum Jannat, Jianying Han, Matilda Houston, Tin Mak, John Watson, Anthony Watson, Paul W. Marshall, Miaomiao Liu, Ronald J. Quinn

**Affiliations:** 1 Institute for Biomedicine and Glycomics, Griffith University, Gold Coast, QLD 4215, Australia; tabassum.jannat@griffithuni.edu.au (T.J.); matilda.houston@griffithuni.edu.au (M.H.); t.mak@griffith.edu.au (T.M.); 2 Institute for Molecular Bioscience, The University of Queensland, Brisbane, QLD 4072, Australia; jianying.han@uq.edu.au; 3 Kimberley Land Council, 11 Gregory St, Broome, WA 6725, Australia; john.watson@klc.org.au (J.W.); anthony.watson@klc.org.au (A.W.); 4 School of Regulation and Global Governance (RegNet), College of Law, Policy and Governance, Australian National University, Canberra, ACT 2600, Australia; paul.marshall1@anu.edu.au

**Keywords:** Mudjala, *Barringtonia acutangula*, bartogenic acid, C-28 glucosides

## Abstract

Mudjala bark, prepared from *Barringtonia acutangula*, is an Australian Aboriginal medicine traditionally used for pain relief, and previous studies have associated its activity with triterpenoid-rich fractions. However, the low-molecular-weight triterpenoids, which were major chromatographic constituents in these fractions, remained chemically unresolved. In this study, we define the aglycone-rich triterpenoid profile of Australian Mudjala bark and resolve its dominant inseparable aglycone mixture. The two major chromatographic isomeric acidic triterpenes could not be separated directly by reversed-phase HPLC, but isolation and hydrolysis of their corresponding C-28 glucosides enabled their assignment as bartogenic acid (**1**) and trachelosperogenin A (**2**). Overall, thirteen triterpenoids were assigned, including one new acylated barringtogenol C-type aglycone (**5**) and twelve known compounds (**1**–**4**, **6**–**13**). The profile comprises three main chemical domains: 24,28-dioic acid aglycones and C-28 glucosides, acylated barringtogenol C-type oleananes, and common pentacyclic triterpene acids. These findings establish a compound-level chemical framework for the previously bioactive low-molecular-weight Mudjala fraction and support future quantitative and biological investigation.

## 1. Introduction

*Barringtonia acutangula* (L.) Gaertn. (Lecythidaceae) is a medicinal tree distributed throughout tropical Asia and northern Australia [1]. The species has a long history of traditional medicinal use across its geographical range and has been used to treat pain, gastrointestinal disorders, respiratory diseases, inflammation, and other ailments [2,3,4]. In the Kimberley region of Western Australia, the bark of *B. acutangula*, locally known as Mudjala, has been used for generations by the Nyikina Mangala Traditional Owners as a remedy for pain and wound management [3,4,5,6]. This traditional use was brought to wider scientific attention following a well-documented case in which Nyikina elder John Watson used the bark for pain relief after a severe crocodile injury [7]. The continued use of Mudjala provides a strong ethnomedical rationale for investigating the chemical constituents that may contribute to its medicinal properties.

Our previous work on Australian Mudjala bark demonstrated potent analgesic activity in bark extracts and chromatographic fractions, leading to the isolation of a series of triterpenoid saponins, acutangulosides A–F [8]. These high-molecular-weight saponins are based on barringtogenol C-type aglycone cores and display diverse glycosylation and acylation patterns. In the same study, a major low-molecular-weight triterpenoid fraction was also obtained and assigned as bartogenic acid. However, the individual constituents of this fraction were not fully resolved, leaving the major chromatographic aglycone composition of Mudjala bark incompletely defined.

More broadly, phytochemical investigations of *B. acutangula* over the past several decades have established this species as a rich source of pentacyclic triterpenoids and triterpenoid saponins (Figure 1) [8,9,10,11,12,13]. Earlier studies on Indian collections, particularly fruits, wood, leaves, and seeds, reported highly oxygenated oleanane-type sapogenins such as barringtogenols B–E, together with triterpene acids including barrigenic, barrinic, tangulic, and acutangulic acids [9,14,15,16,17,18,19,20]. These compounds are characterised by extensive oxygenation of the oleanane skeleton, frequently involving hydroxy, carboxylic acid, acyl, or glycosidic substitutions at the C-3, C-16, C-21, C-22, C-23/C-24, and C-28 positions. More recent studies have also identified ursane- and oleanane-type acids and glycosides, including ilexsaponin A1, ilexosapogenin A, arjunic acid, tormentic acid, and taraxerol, from *B. acutangula* flower and leaf extracts [12,13]. In addition, seed and bark studies have revealed larger triterpenoid saponins, including barringtosides and acutangulosides, many of which are based on barringtogenol C-type aglycone cores [8,11].

In the present study, we investigated the low-molecular-weight triterpenoid fraction of Australian Mudjala bark. Thirteen triterpenoids, including one new acylated oleanane-type aglycone and twelve known compounds, were isolated or assigned using LC–MS, HRESIMS, and comprehensive one- and two-dimensional NMR analyses. Particular attention was given to resolving the major chromatographic constituents present as an inseparable aglycone mixture through isolation and hydrolysis of the corresponding C-28 glucosides. These results establish a compound-level triterpenoid profile for Australian Mudjala bark and provide a chemical foundation for future authentication, quantitative analysis, and pharmacological investigation of this culturally important medicinal plant.

## 2. Results

### 2.1. Chemical Resolution of an Inseparable Triterpene Mixture

During our previous phytochemical investigation of Mudjala bark, a triterpenoid aglycone fraction was isolated and reported as bartogenic acid [8]. Re-examination of the original NMR data, however, revealed two sets of closely related resonances, accounting for approximately 92% and 8% of the mixture, respectively (Figure S1). The predominant resonances supported assignment of the major oleanane component as bartogenic acid, whereas the identity of the minor constituent remained unresolved. To clarify the composition of this fraction and establish the major chromatographic constituents of the Mudjala aglycone profile, we reinvestigated the low-molecular-weight triterpenoid fraction.

The analytical LC-MS trace had one major peak which was in the expected *m*/*z* range of triterpenoids, designated as **1**. LC–MS analysis of the fraction revealed an isomer of the major chromatographic triterpenoid **1** with an earlier retention time, designated as **2** (Figure 2A), accounting for an approximately 7:3 ratio in the crude extract, respectively. Both exhibited identical molecular ions at *m*/*z* 517.3168 [M − H]^−^, corresponding to the molecular formula C_30_H_46_O_7_ and indicating that they were isomeric aglycones. The difference from the relative abundance observed in the earlier material may reflect variation in the plant collections and extraction procedures, as the previous fraction was obtained from an aqueous extract of bark collected in 1989 and 1994, whereas the present fraction originated from a 95% ethanolic extract of bark collected in 2013.

Repeated reverse-phase HPLC under multiple solvent systems failed to resolve the two isomers on a preparative scale (Figure 2B). In contrast, two earlier-eluting constituents, **3** and **4**, were resolved, and both gave deprotonated molecular ions at *m*/*z* 679.3724 [M − H]^−^, 162 Da higher than those of **1** and **2**, consistent with hexosylated analogues (Figure 2C). Comparison of their HRESIMS and NMR data with published values identified **3** as 28-O-β-D-glucopyranosyl bartogenic acid [21] and **4** as trachelosperoside A1 [22,23] (Figure 3). Compound **3** has been isolated from several plant species, but the published data comprise only the ^13^C and some key ^1^H NMR data [21]; therefore, we have included the complete 1D and 2D NMR data for **3** in the Supplementary Information (Table S1).

The isolation of **3** and **4** provided a strategy to resolve the identities of the unresolved aglycones. Since **3** and **4** were established as C-28 glucosyl esters and differed from **1** and **2** by C_6_H_10_O_5_, alkaline hydrolysis was used to cleave the ester-linked glucose unit. The hydrolysis products were then compared directly with the original aglycone mixture by LC–MS and ^1^H NMR (Figure 4).

LC–MS analysis showed the expected loss of 162 Da from the glucosides, giving hydrolysis products with ions at *m*/*z* 517.3168 [M − H]^−^ (calc. 517.3165; absolute mass error 0.0003 Da) and retention times (t_R_ of hydrolysed **3** = 17.4 min, t_R_ of hydrolysed **4** = 16.0 min) matching the corresponding aglycones in the original mixture (t_R_ of **1** = 17.4 min, t_R_ of **2** = 16.0 min). Hydrolysis of the oleanane glucoside **3** afforded an aglycone matching compound **1**, while hydrolysis of the ursane glucoside **4** afforded an aglycone matching compound **2**.

The assignments were further supported by diagnostic ^1^H NMR resonances. The hydrolysis product of **3** showed the characteristic features of compound **1**, including the olefinic proton at δ_H_ 5.23 ppm and the coupled H-18/H-19 resonances at δ_H_ 2.92 and 3.12 ppm. Together with comparison to reported NMR data [24], these results confirmed compound **1** as 2α,3β,19α-trihydroxyolean-12-ene-24,28-dioic acid, also known as bartogenic acid.

Similarly, the hydrolysis product of **4** showed the diagnostic features of compound **2**, including the olefinic proton at δ_H_ 5.18 ppm, the H-18 broad singlet at δ_H_ 2.37–2.38, and a methyl doublet characteristic of the rearranged ursane E ring. By comparing with literature data [23,25], compound **2** was consequently identified as 2α,3β,19α-trihydroxyurs-12-ene-24,28-dioic acid, also known as trachelosperogenin A (Figure 4).

This hydrolysis-based strategy resolved the long-standing assignment problem for the two major chromatographic aglycones in Mudjala, bartogenic acid (**1**) and trachelosperogenin A (**2**). Access to the individual aglycones now enables independent biological evaluation, structure–activity studies, and quantitative profiling of the two major chromatographic triterpenes, which was not possible when they were available only as an inseparable mixture.

### 2.2. Characterisation of a New Acylated Oleanane Triterpenoid

Chemical investigation of the low-molecular-weight fraction of the ethanolic extract of Mudjala bark also led to the isolation of a new triterpenoid aglycone (**5**). Its structure was established by comprehensive 1D and 2D NMR analyses, including COSY, HSQC, HMBC, and ROESY experiments, together with HRESIMS (Table 1, Figures S2–S6).

Compound **5** was obtained as a white crystal. Its molecular formula was established as C_42_H_60_O_7_ from the HRESIMS ion at *m*/*z* 699.4235 [M + Na]^+^ (calcd for C_42_H_60_O_7_Na, 699.4237), corresponding to 13 degrees of unsaturation. The ^1^H NMR spectrum displayed features of an oxygenated oleanane-type triterpenoid, including seven tertiary methyl singlets at δ_H_ 1.44, 1.07, 0.91, 0.88, 0.85, 0.82, and 0.69 ppm; two downfield methyl multiplets at δ_H_ 1.60 (dq, J = 7.2, 1.2 Hz) and 1.52 (q, J = 1.2 Hz); two olefinic protons at δ_H_ 6.60 (qq, J = 7.2, 1.2 Hz) and 5.32 (t, J = 3.7 Hz); seven methines, including four oxygenated methine protons at δ_H_ 6.01 (d, J = 10.2 Hz), 5.64 (d, J = 10.2 Hz), 3.98 (dd, J = 6.7, 3.6 Hz), and 3.01 (dd, J = 11.1, 5.0 Hz); and eight methylenes, including an oxygenated methylene at δ_H_ 3.07 (d, J = 10.2 Hz) and 2.82 (d, J = 10.2 Hz). In addition, five aromatic proton signals at δ_H_ 7.85 (2H, dd, J = 8.2, 1.2 Hz), 7.61 (1H, dddd, J = 8.2, 7.4, 1.2, 1.2 Hz), and 7.49 (2H, dd, J = 8.2, 7.4 Hz) indicated the presence of a monosubstituted benzoyl group. Three exchangeable hydroxy protons were observed at δ_H_ 4.89, 4.46, and 4.31. The carbon chemical shifts, assigned from HSQC and HMBC data, accounted for 42 carbon resonances. Characteristic signals included two ester carbonyl carbons at δ_C_ 167.4 and 166.1; six aromatic carbons at δ_C_ 133.0, 130.8, 129.1 (2C), and 128.4 (2C); an additional four non-aromatic olefinic carbons at δ_C_ 142.3, 136.5, 129.3 and 123.4; four oxygenated methine carbons at δ_C_ 79.4, 77.1, 73.0 and 68.1; and one oxygenated methylene carbon at δ_C_ 63.0. These data suggested a highly oxygenated oleanane-type triterpenoid bearing two acyl substituents, one of which was aromatic.

The COSY spectrum established several key proton spin systems, including an oxygenated methine pair at δ_H_ 6.01/5.64, an olefinic methine/methyl fragment at δ_H_ 6.60/1.52, and the aromatic proton sequence of a monosubstituted benzene ring. The latter, together with the aromatic carbon resonances and an ester carbonyl carbon at δ_C_ 166.1, established a benzoyl group. The second acyl unit was assigned as a tigloyl group based on the olefinic proton at δ_H_ 6.60, two methyl resonances at δ_H_ 1.52 and 1.60, and HMBC correlations from these protons to the olefinic carbons and carbonyl carbon at δ_C_ 167.4. The methyl carbon shifts at δ_C_ 12.5 and 14.4 supported the assignment of this unit as an E-configured tigloyl group rather than the corresponding Z-configured angeloyl isomer.

The oleanane framework was established from the remaining COSY and HMBC correlations. The triterpenoid olefinic proton at δ_H_ 5.32 showed HMBC correlations to δ_C_ 36.5, 46.9, and 41.8, supporting a Δ12 oleanane skeleton. HMBC correlations from the oxygenated methine proton at δ_H_ 3.01 to the methyl carbon at δ_C_ 16.6 supported oxygenation at C-3. The oxygenated methine proton at δ_H_ 3.98 showed ROESY correlations with adjacent methylene and methyl protons, consistent with the C-16 hydroxy-bearing centre of a barringtogenol C-type skeleton. The coupled oxygenated methine protons at δ_H_ 6.01 and 5.64 defined the C-21/C-22 spin system, and their large coupling constant (J = 10.2 Hz) supported a fixed trans relationship across this unit.

The positions of the two acyl groups were determined by key HMBC correlations from the oxygenated methine protons to the corresponding ester carbonyl carbons. The HMBC correlation from the proton at δ_H_ 6.01 to the benzoyl carbonyl carbon at δ_C_ 166.1 located the benzoyl group at C-21, while the HMBC correlation from the proton at δ_H_ 5.64 to the tigloyl carbonyl carbon at δ_C_ 167.4 established the tigloyl group at C-22. Together, these data identified compound **5** as 21-O-benzoyl-22-O-tigloylbarringtogenol C (Figure 5).

ROESY correlations between H-3/H-5/H-23, H-27/16-OH, and H-12/H-18/H-30 (Table 1) enabled the determination of the relative configuration of compound **5**. The ROESY spectrum confirmed spatial relationships consistent with those of previously described analogues, e.g., chakasapogenin II (**6**). The absolute stereochemistry was corroborated by an optical rotation value of ${\left[\alpha \right]}_{D}^{21}$ + 73.0 (c 0.2, MeOH), which correlates in direction with the reported literature values. Therefore, the absolute stereochemistry of compound **5** was concluded to be identical to previously established configurations sharing this core scaffold.

### 2.3. Triterpenoid Profile of Mudjala Bark

In addition to the two major chromatographic aglycones **1** and **2**, their C-28 glucosides **3** and **4**, and the new triterpenoid aglycone **5**, eight known triterpenoids and triterpenoid glycosides were isolated and identified by comparison of their HRESIMS and NMR data with reported values. These compounds comprised chakasapogenin II (**6**) [26]; the ursane-type glycosides chestnoside B (**7**) [27] and ilexsaponin A1 (**8**) [28]; and the oleanane-type barringtogenol C derivatives cyrillin A (**9**) [29], bemeuxin (**10**) [30], and barringtogenol E (**11**) [31], together with the common pentacyclic triterpenes betulinic acid (**12**) [32] and ursolic acid (**13**) [33] (Figure 6). Because detailed modern NMR spectroscopic data for bemeuxin (**10**) are limited in the literature, complete 1D and 2D NMR data for **10** are provided in the Supplementary Information (Table S2).

LC–MS analysis of the triterpenoid fraction provided an overview of the aglycone-rich chemical profile of Mudjala bark (Figure 7). Thirteen compounds were assigned within this profile, defining the major triterpenoid space of the bark extract and providing a complementary profile to the previously reported high-molecular-weight acutanguloside saponins [8].

The most prominent feature of this profile was the paired occurrence of acidic triterpene aglycones and their C-28 glucosides. Compounds **1**–**4**, **7**, and **8** belong to a 24,28-dioic acid triterpenoid class, in which the aglycone core contains a C-24 carboxylic acid and either a free C-28 carboxylic acid or a C-28 glucosyl ester. In contrast, compounds **5**, **6**, and **9**–**11** belong to a barringtogenol C-type oleanane class. These compounds lack the C-24/C-28 diacid motif and are characterised by a highly oxygenated oleanane core with substitution around C-16, C-21, C-22, and C-28. Compounds **5** and **6** extend this class through tigloyl and benzoyl/tigloyl acylation patterns. Betulinic acid (**12**) and ursolic acid (**13**) represent a third, less oxygenated triterpene acid class, containing a single C-28 carboxylic acid and lacking both the 24,28-dioic acid motif and the barringtogenol C-type polyoxygenation pattern.

This profile is chemically distinct from the acutanguloside-type saponins previously reported from *B. acutangula*. Those saponins are based primarily on a barringtogenol C-type aglycon and contain larger sugar chains attached through C-3, with additional acylation or glycosylation patterns depending on the individual compound. In contrast, compounds **3** and **4** are simple C-28 glucosyl esters of acidic oleanane and ursane aglycones. Thus, the major C-28 glucosides in the LC-MS chromatogram identified here are not direct glycosylated forms of the previously reported acutanguloside saponins but represent a separate branch of Mudjala triterpenoid chemistry.

## 3. Discussion

The present study establishes the low-molecular-weight triterpenoid profile of Australian Mudjala bark and resolves a long-standing chemical ambiguity associated with its major triterpenoid fraction. Previous biological studies and patent disclosures demonstrated potent analgesic activity in Mudjala extracts and fractions [34], but the major chromatographic triterpenoid constituents of the active low-molecular-weight fraction could not be resolved as individual compounds. In the present work, the isolation of the corresponding C-28 glucosides **3** and **4**, followed by hydrolysis, enabled the assignment of the inseparable aglycones **1** and **2** as bartogenic acid and trachelosperogenin A, respectively. The NMR data (Tables S1, S3 and S4) of the C-28 glucosides and their hydrolysed products confirmed that the original aglycone core was retained after the alkaline hydrolysis. This resolution is significant because it defines the two major chromatographic constituents at the compound level, providing a chemical basis for future biological evaluation, quantitative analysis, and structure–activity studies.

A key feature of the Mudjala profile is the organisation of the major chromatographic low-molecular-weight constituents around paired acidic aglycones and their C-28 glucosides. Compounds **1** and **2** were the dominant free aglycones, while compounds **3** and **4** were their matched glycosylated forms, respectively. Together with the additional C-28 glycosides **7** and **8**, these compounds define a 24,28-dioic acid triterpenoid domain within the bark extract. This domain is chemically distinct from the barringtogenol C-type oleanane domain represented by the new compound **5** and the known analogues **6**, **9**–**11**, which lack the C-24/C-28 diacid motif and instead show the polyoxygenated and acylated features typical of barringtogenol C-related triterpenes.

This distinction is relevant to the broader chemistry of *B. acutangula*. Previous work on Australian Mudjala bark emphasised high-molecular-weight acutanguloside saponins, which are based on barringtogenol C-type aglycones bearing larger C-3 sugar chains [8]. In contrast, the major glycosides in the LC-MS chromatogram that are characterised here, **3** and **4**, are simple C-28 glucosyl esters of acidic oleanane and ursane aglycones. The coexistence of these two chemical domains may be important for future authentication and quality control.

The identification of the new acylated oleanane-type aglycone **5** further expands the known triterpenoid diversity of Mudjala. The benzoyl/tigloyl substitution pattern of compound **5** indicates that the acylation chemistry previously observed in Mudjala saponins also extends to low-molecular-weight aglycones. In addition, several known compounds identified in this study are reported from *B. acutangula* for the first time, including compounds **2**, **3**, **4**, **6**, **7**, **9**, **10**, **12**, and **13**. These findings define a structured low-molecular-weight triterpenoid profile in Australian Mudjala bark, comprising acidic aglycone/C-28 glucoside pairs as the predominant LC-MS feature, acylated barringtogenol C-type aglycones, and additional pentacyclic triterpene acids.

Historically, phytochemical characterisation of *B. acutangula* (Lecythidaceae) has focused heavily on specimens harvested across South Asia, where the plant serves as a cornerstone of traditional Ayurvedic formulations for gastrointestinal, metabolic, and inflammatory ailments. These investigations have consistently yielded broad-spectrum metabolites, including triterpenoid saponins, gallic acid, and common flavonoid glycosides. However, the Australian geographic variant reveals a distinct biosynthetic divergence. The profiling of Australian *B. acutangula* has led to the isolation and structural elucidation of nine novel compounds previously undocumented in the Indian chemotype. The major new entities contain a 1,2 –methyl shift in the E ring. This distinct secondary metabolite profile underscores evolutionary adaptation to the Australian ecosystem, aligning closely with its unique ethnobotanical utility as a potent native analgesic. The identification of these nine novel entities establishes the Australian variety not merely as a geographic variant but as a structurally distinct chemical repository with targeted therapeutic potential separate from the classic South Asian archetype. A summary of the new and previously identified triterpenoids is presented in Table 2.

The resulting profile provides a foundation for future studies aimed at determining the individual biological activities of the major chromatographic aglycones and glycosides, developing quantitative markers for Mudjala authentication and quality control, and understanding how the low-molecular-weight triterpenoids complement the previously reported acutanguloside saponins in this culturally important medicinal plant.

## 4. Materials and Methods

### 4.1. General Experimental Procedures

NMR experiments were conducted in DMSO-*d*_6_ (δ_H_ 2.50, δ_C_ 39.5) or CDCl_3_ (δ_H_ 7.26, δ_C_ 77.2) at 25 °C on a Bruker Avance III HDX 800 MHz NMR spectrometer equipped with a TCI cryoprobe (Bruker BioSpin GmbH, Rheinstetten, Germany). HRESIMS and LC–MS data were acquired on a Bruker maXis II ESI-QTOF mass spectrometer (Bruker BioSpin GmbH, Rheinstetten, Germany) coupled to a Thermo Fisher Scientific UltiMate 3000 UHPLC system (ThermoFischer Scientific, Waltham, MA, USA). LC–MS analyses were performed using a Phenomenex Luna C18 column (3 μm, 2 × 150 mm) (Phenomenex Inc., Torrance, CA, USA) maintained at room temperature with an injection volume of 1 μL and a flow rate of 0.3 mL/minute. Injection concentrations for the LC-MS analyses were 5 ppm for pure compounds and 50 ppm for mixtures and crude extracts. The MS instrument was calibrated using the manufacturer’s calibration standards. The scan was collected in an *m*/*z* range of 400–1200 amu. The *m*/*z* of each identified compound was calculated to 4 decimal places and measured with a mass accuracy of <3.5 ppm. The capillary voltage was 4.0 kV, the end plate offset was 500 V, the dry temperature was set at 220 °C, and the dry gas flow was set at 4.0 L/min. The transfer time was set to 80 μs. HPLC analyses and purifications were performed using Thermo Fisher Scientific UltiMate 3000 HPLC systems with Phenomenex Onyx Monolithic C18 (100 × 4.6 mm, 5 μm) (Phenomenex Inc., Torrance, CA, USA), Thermo Fisher Scientific Betasil C18 (100 × 4.6 mm, 5 μm; 150 × 10 mm, 5 μm; 250 × 10 mm, 5 μm), and Phenomenex Luna C18 (150 × 10 mm, 5 μm) columns. Optical rotations were measured on a JASCO P-2000 polarimeter (JASCO Corporation, Tokyo, Japan). All the NMR analysis was done with MastreNova (v14.3.2), HPLC software used was Chromeleon 7.2 and LC-MS analysis software was Bruker Compass DataAnalysis.

### 4.2. Plant Material

Mudjala bark was collected from the Kimberley region of Western Australia. The identity of the tree was confirmed as *Barringtonia acutangula* var. *acutangula*, and a voucher (#AQ595349) was deposited with the Queensland Herbarium in 2005. The dried bark material (~30 kg) was extracted twice with 95% ethanol/water (50 L) to afford the crude ethanolic extract (~1 kg), which was used for subsequent fractionation and isolation.

### 4.3. Extraction and Fractionation

The crude ethanolic extract (3 × 100 g) was subjected to Sephadex LH-20 size-exclusion chromatography (3.1 L 95% ethanol), and the low-molecular-weight fraction was selected for triterpenoid isolation. This fraction (~65 g) was further separated by reversed-phase C18 flash chromatography using a step gradient of MeOH/H_2_O (65%, 90%, and 100% MeOH; bed volume 500 mL) to give two main fractions, F1 and F2. Fraction F1 (~10 g) contained the more polar triterpenoid acids and glycosides, including the major chromatographic constituents **1** and **2** and their C-28 glucosides **3** and **4**. Fraction F2 (~3 g) contained less polar aglycones, including acylated oleanane-type triterpenoids.

### 4.4. Isolation of Major Chromatographic Aglycones and C-28 Glucosides

Fraction F1 (10 g) was subfractionated by semipreparative reversed-phase HPLC using a Betasil C18 column (ThermoFischer Scientific, Waltham, MA, USA) with a gradient from 50% MeOH/H_2_O to 100% MeOH over 75 min at 7 mL/min to afford subfractions F1.1–F1.5. Subfractions F1.1 and F1.2 contained the C-28 glucosides **3** and **4** together. These compounds were purified by reversed-phase HPLC using a Luna C18 column (5 μm, 10 × 150 mm) under isocratic 25% MeCN/H_2_O at 4 mL/min to afford compound **3** (28-O-glucosylbartogenic acid, t_R_ 14.5 min, 10.0 mg) and compound **4** (trachelosperoside A1, t_R_ 16.4 min, 4.0 mg).

Subfractions F1.3–F1.5 contained the major chromatographic constituents **1** and **2** as an inseparable mixture with chestnoside B (**7**). Analytical LC–MS using a Luna C18 column (3 μm, 2 × 150 mm) under dilute sample conditions and isocratic 35% MeCN/H_2_O (0.1% formic acid as additive) showed partial separation of the two isomers, but repeated preparative reversed-phase HPLC failed to resolve them on a larger scale. The aglycone mixture (t_R_ 10 min, 2.6 mg) and compound **7** (chestnoside B, t_R_ 13 min, 0.5 mg) were therefore collected by HPLC using a Luna C18 column (5 μm, 10 × 150 mm) with isocratic 35% MeCN/H_2_O at 4 mL/min.

### 4.5. Alkaline Hydrolysis of C-28 Glucosides ***3*** and ***4***

Compounds **3** and **4** were individually hydrolysed to generate the corresponding aglycones. Each compound (5 mg) was dissolved in 200 mM KOH (5 mL) and heated at 80 °C for 2 h under argon. The reaction mixture was neutralised with 37% HCl and extracted with ethyl acetate and water. The ethyl acetate fraction was washed three times with deionised water, concentrated under reduced pressure, and freeze-dried to yield approximately 3 mg of each aglycone product. The hydrolysis products were analysed by LC–MS using a Luna C18 column (3 μm, 2 × 150 mm) with isocratic 35% MeCN/H_2_O, and their retention times and mass spectra were compared with those of the original aglycone mixture.

### 4.6. Isolation of New Acylated Aglycone ***5*** and Additional Known Triterpenoids (***6***–***13***)

Fraction F2 (3 g) was separated by semipreparative reversed-phase HPLC using a Betasil C18 column with a gradient from 50% MeOH/H_2_O to 100% MeOH over 75 min at 7 mL/min to afford five subfractions, F2.1–F2.5. Crystalline material obtained from these fractions was further purified by semipreparative reversed-phase HPLC using a Betasil C18 column (5 μm, 10 × 150 mm) with isocratic 68% MeOH/H_2_O at 4 mL/min to yield cyrillin A (**9**, t_R_ 22.2 min, 1.3 mg), bemeuxin (**10**, t_R_ 23.9 min, 3.7 mg), and chakasapogenin II (**6**, t_R_ 27.4 min, 2.8 mg).

A second isolation was performed from 15 g of crude Mudjala extract. The extract was fractionated by Sephadex LH-20 chromatography (3.1 L) using MeOH to afford 13 fractions (I–XIII). Fraction IV (400 mg) was subfractionated by HPLC using a Betasil C18 column (5 μm, 10 × 250 mm) with a gradient from 50% MeOH/H_2_O to 100% MeOH over 50 min, and fractions were collected at 1 min intervals. Subfraction IV-22 was further purified by semipreparative Betasil C18 HPLC using a slow gradient from 70% to 80% MeOH/H_2_O at 3 mL/min to afford ilexsaponin A1 (**8**, t_R_ 14.0 min, 0.65 mg). Subfraction IV-38 was purified by isocratic 85% MeOH/H_2_O using the same column and flow rate to afford barringtogenol E (**11**, t_R_ 27.1 min, 1.0 mg) and the new compound 21-O-benzoyl-22-O-tigloylbarringtogenol C (**5**, t_R_ 28.5 min, 1.1 mg). Subfraction IV-41 yielded betulinic acid (**12**, t_R_ 41.0 min, 11.0 mg), and subfractionation of IV-43 under isocratic 90% MeOH/H_2_O afforded ursolic acid (**13**, t_R_ 22.3 min, 0.93 mg).

### 4.7. Spectroscopic Data

Spectroscopic data for compounds **1**–**13** are summarised below. Complete ^1^H and ^13^C NMR assignments and comparisons with literature data are provided in the Supplementary Information.

Bartogenic acid (**1**): ${\left[\alpha \right]}_{D}^{25}$ + 27.0 (*c* 0.1, MeOH); HRESIMS *m*/*z* 517.3168 [M − H]−, (calcd for C_30_H_46_O_7_, 517.3165; calc. 518.3241); for ^1^H and ^13^C NMR data, see Tables S3 and S4.

Trachelosperogenin A (**2**): ${\left[\alpha \right]}_{D}^{25}$ + 39.0 (*c* 0.2, MeOH); HRESIMS *m*/*z* 517.3168 [M − H]^−^, (calcd for C_30_H_45_O_7_, 517.3165); for ^1^H and ^13^C NMR data, see Tables S3 and S4.

28-O-β-D-Glucopyranosylbartogenic acid (**3**): white amorphous powder; ${\left[\alpha \right]}_{D}^{25}$ + 41.0 (*c* 0.08, MeOH); HRESIMS *m*/*z* 679.3724 [M − H]^−^, (calcd for C_36_H_56_O_12_, 679.3674; for ^1^H and ^13^C NMR data, see Table S1.

Trachelosperoside A1 (**4**): white amorphous powder; ${\left[\alpha \right]}_{D}^{25}$ + 12.5 (*c* 0.5, MeOH); HRESIMS *m*/*z* 679.3724 [M − H]^−^, (calcd for C_36_H_55_O_12_, 679.3694); for ^1^H and ^13^C NMR data, see Tables S3 and S4.

21-O-benzoyl-22-O-tigloylbarringtogenol C (**5**): white crystals; ${\left[\alpha \right]}_{D}^{21}$ + 73.0 (*c* 0.2, MeOH); HRESIMS *m*/*z* 699.4235 [M + Na]^+^, (calcd for C_42_H_60_O_7_Na, 699.4237; for ^1^H and ^13^C NMR data, see Table 1.

Chakasapogenin II (**6**): white crystals; ${\left[\alpha \right]}_{D}^{25}$ + 59.0 (*c* 0.5, MeOH); HRESIMS *m*/*z* 617.4104 [M + HCOO]^−^, (calcd for C_35_H_56_O_6_HCOO, 617.4154); for ^1^H and ^13^C NMR data, see Tables S3 and S4.

Chestnoside B (**7**): white amorphous powder; ${\left[\alpha \right]}_{D}^{25}$ + 15.1 (*c* 0.08, MeOH); HRESIMS 661.3569 [M − H]^−^, (calcd for C_36_H_53_O_11_, 661.3588); for ^1^H and ^13^C NMR data, see Tables S3 and S4.

Ilexsaponin A1 (**8**): ${\left[\alpha \right]}_{D}^{21}$ + 30.6 (*c* 0.15, MeOH); HRESIMS *m*/*z* 663.3735 [M − H]^−^, (calcd for C_36_H_55_O_11_, 663.3744); for ^1^H and ^13^C NMR data, see Tables S3 and S4.

Cyrillin A (**9**): white amorphous powder; ${\left[\alpha \right]}_{D}^{25}$ + 56.0 (*c* 0.06, MeOH); HRESIMS 509.3238 [M + Na]^+^, (calcd for C_30_H_46_O_5_Na, 509.3243); for ^1^H and ^13^C NMR data, see Tables S3 and S4.

Bemeuxin (**10**): white amorphous powder; ${\left[\alpha \right]}_{D}^{25}$ + 72.0 (*c* 0.05, MeOH); HRESIMS *m*/*z* 533.3420 [M + HCOO]^−^, (calcd for C_30_H_48_O_5_HCOO, 533.3478); for ^1^H and ^13^C NMR data, see Table S2.

Barringtogenol E (**11**): white amorphous powder; ${\left[\alpha \right]}_{D}^{21}$ + 67.0 (*c* 0.2, MeOH); HRESIMS *m*/*z* 721.4060 [M + Na]^+^, (calcd for C_44_H_58_O_7_Na, 721.4081); for ^1^H and ^13^C NMR data, see Tables S3 and S4.

Betulinic acid (**12**): white amorphous powder; ${\left[\alpha \right]}_{D}^{21}$ + 24.7 (*c* 0.3, MeOH); HRESIMS *m*/*z* 479.3477 [M + Na]^+^, (calcd for C_30_H_48_O_3_Na, 479.3501); for ^1^H and ^13^C NMR data, see Tables S3 and S4.

Ursolic acid (**13**): white amorphous powder; ${\left[\alpha \right]}_{D}^{21}$ + 30.4 (*c* 0.5, MeOH); HRESIMS *m*/*z* 455.3503 [M − H]^−^, (calcd for C_30_H_47_O_3_, 455.3525); for ^1^H and ^13^C NMR data, see Tables S3 and S4.

### 4.8. Triterpenoid Profile Development

Ethanolic crude extract was dissolved in 50% ACN/H_2_O to a final concentration of 1 mg/mL. The solution was then sonicated, filtered, and used as a stock solution of crude extract. 50 ppm working solution was prepared from the stock solution. The LC-MS profile was developed using a Phenomenex Luna C18 column (3 μm, 2 × 150 mm) with an initial 6 min isocratic 40% ACN/H_2_O, followed by a gradient from 40% ACN/H_2_O to 99% ACN over 20 min at 0.3 mL/min with an injection volume of 1 μL to afford the total profile.

## Figures and Tables

**Figure 1:**
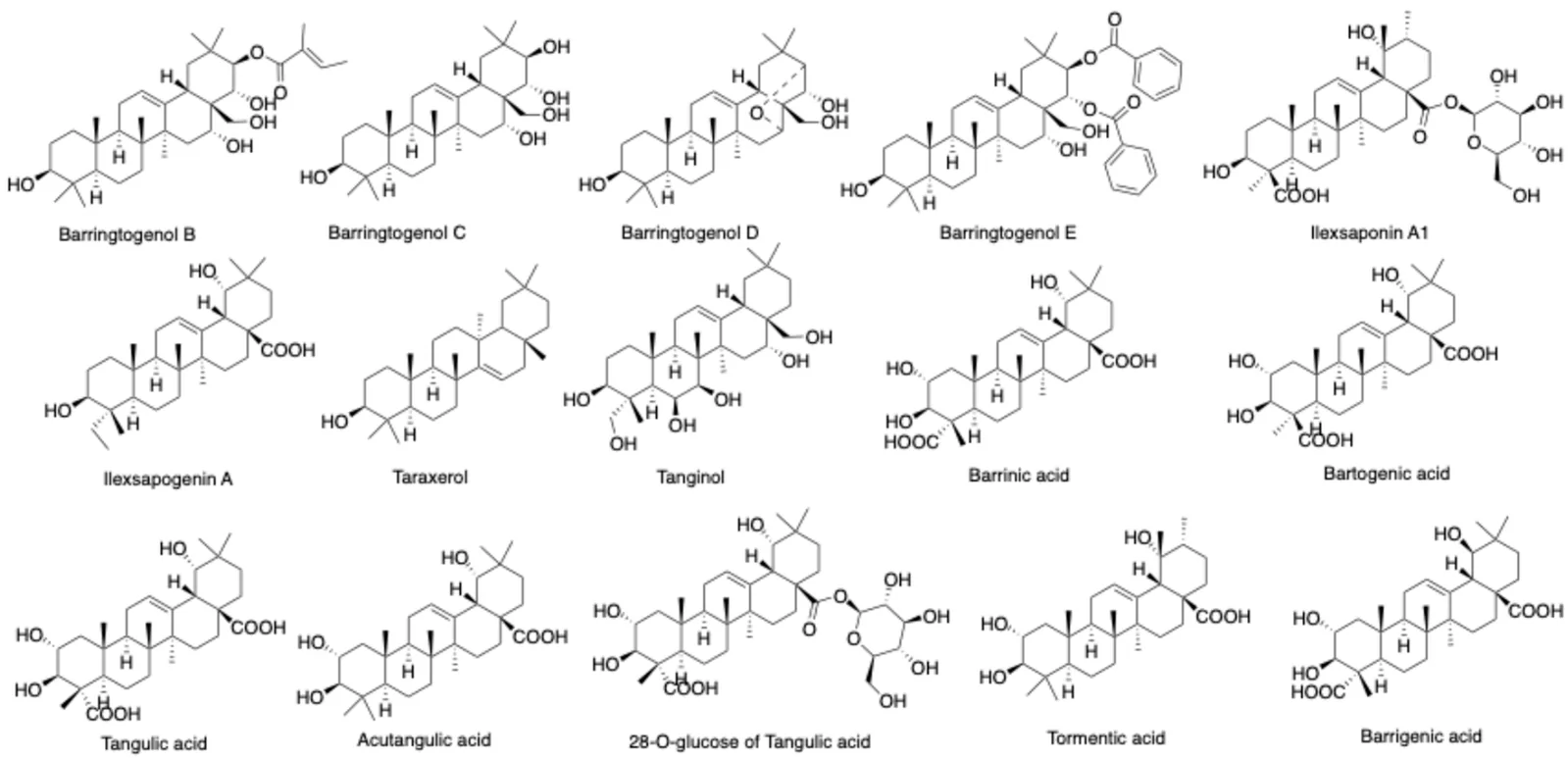
Previously isolated triterpenoid aglycones from *B. acutangula*.

**Figure 2:**
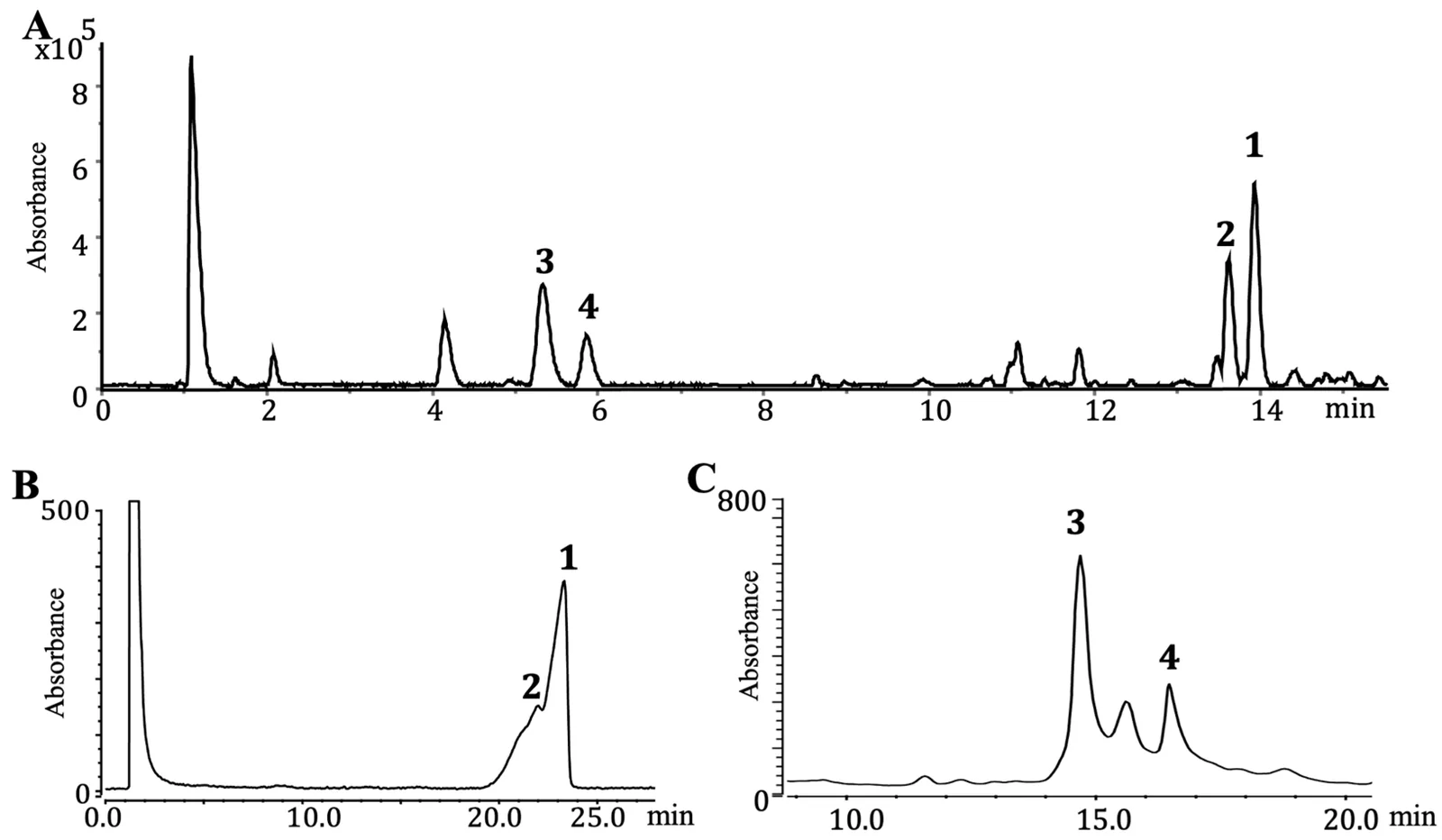
Chemical resolution of the major chromatographic triterpenoid constituents of Mudjala bark through their corresponding C-28 glucosides: (**A**) Analytical LC–MS chromatogram of the low-molecular-weight fraction showing the major triterpenoids in the chromatogram and their glycosides of Mudjala bark. (**B**) Semi-preparative HPLC chromatograms of the aglycone isomers **1** and **2**. (**C**) Isolation of **3** and **4** by semi-preparative HPLC.

**Figure 3:**
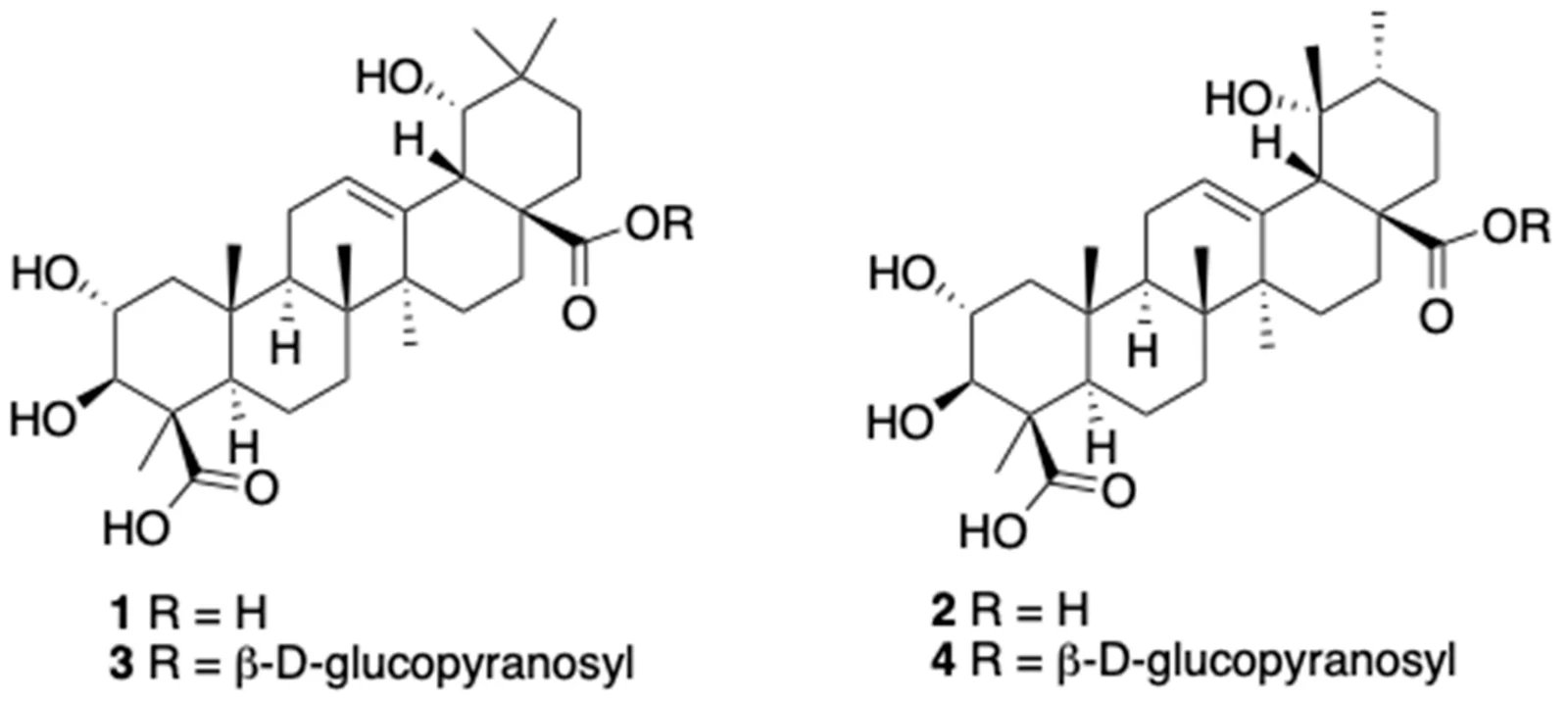
Chemical structures of compounds **1**–**4**.

**Figure 4:**
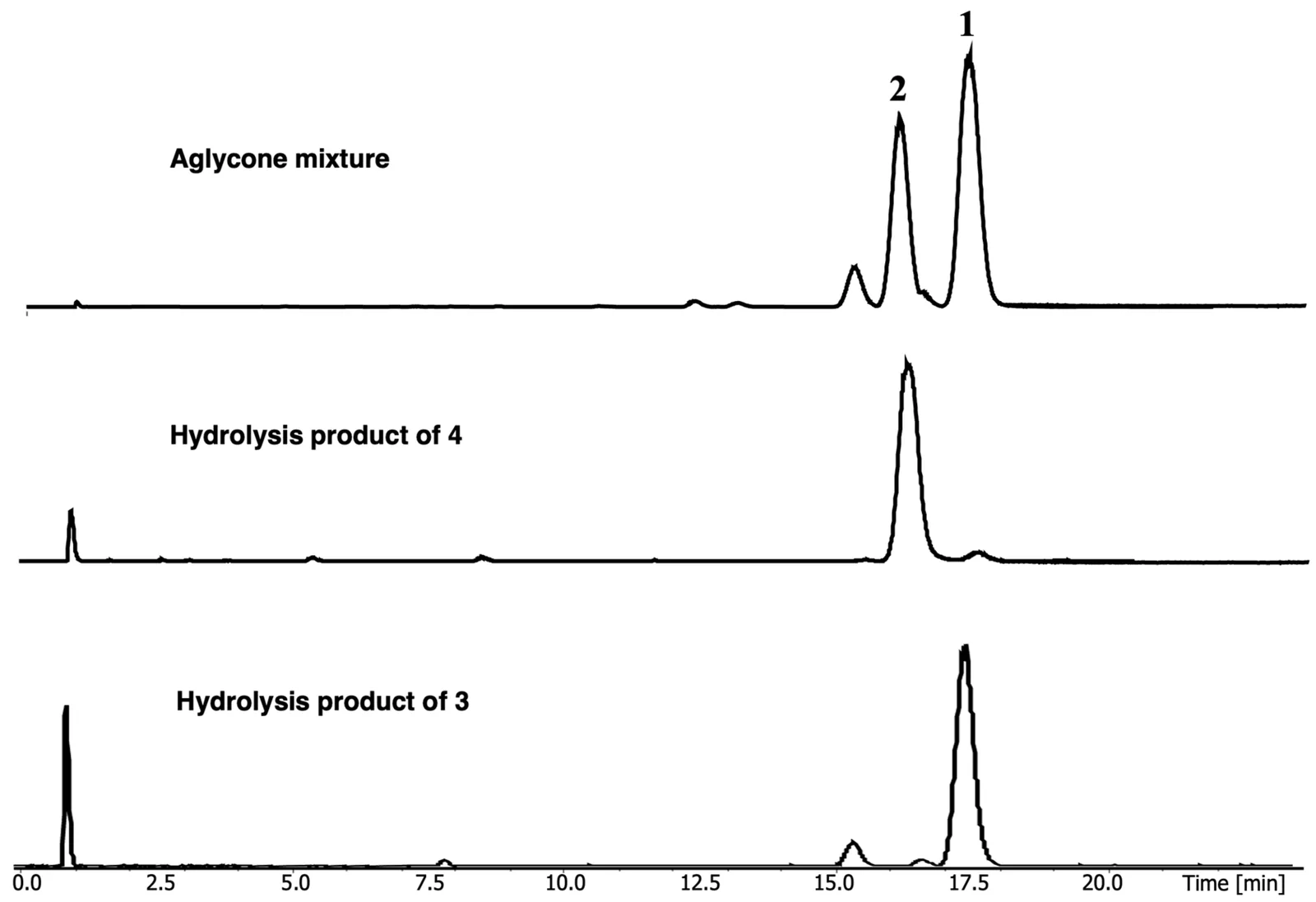
LC–MS comparison of the inseparable aglycone mixture **1**/**2** with the hydrolysis products of glucosides **3** and **4**.

**Figure 5:**
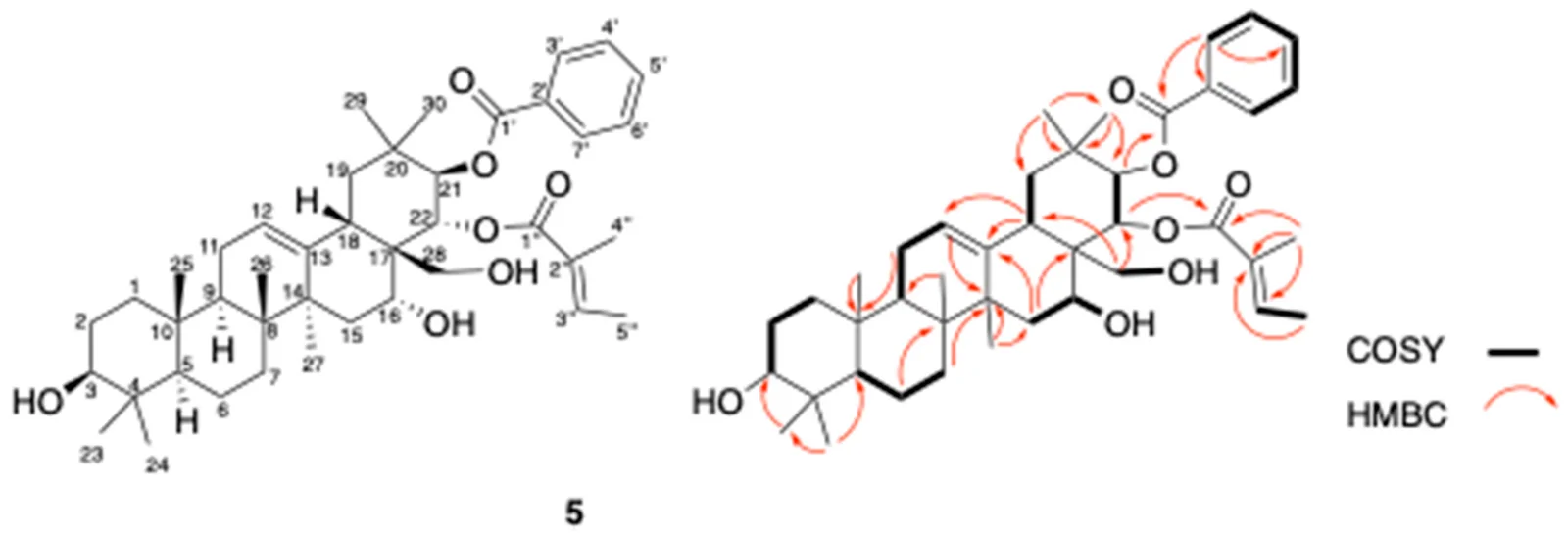
Chemical structure of compound **5**.

**Figure 6:**
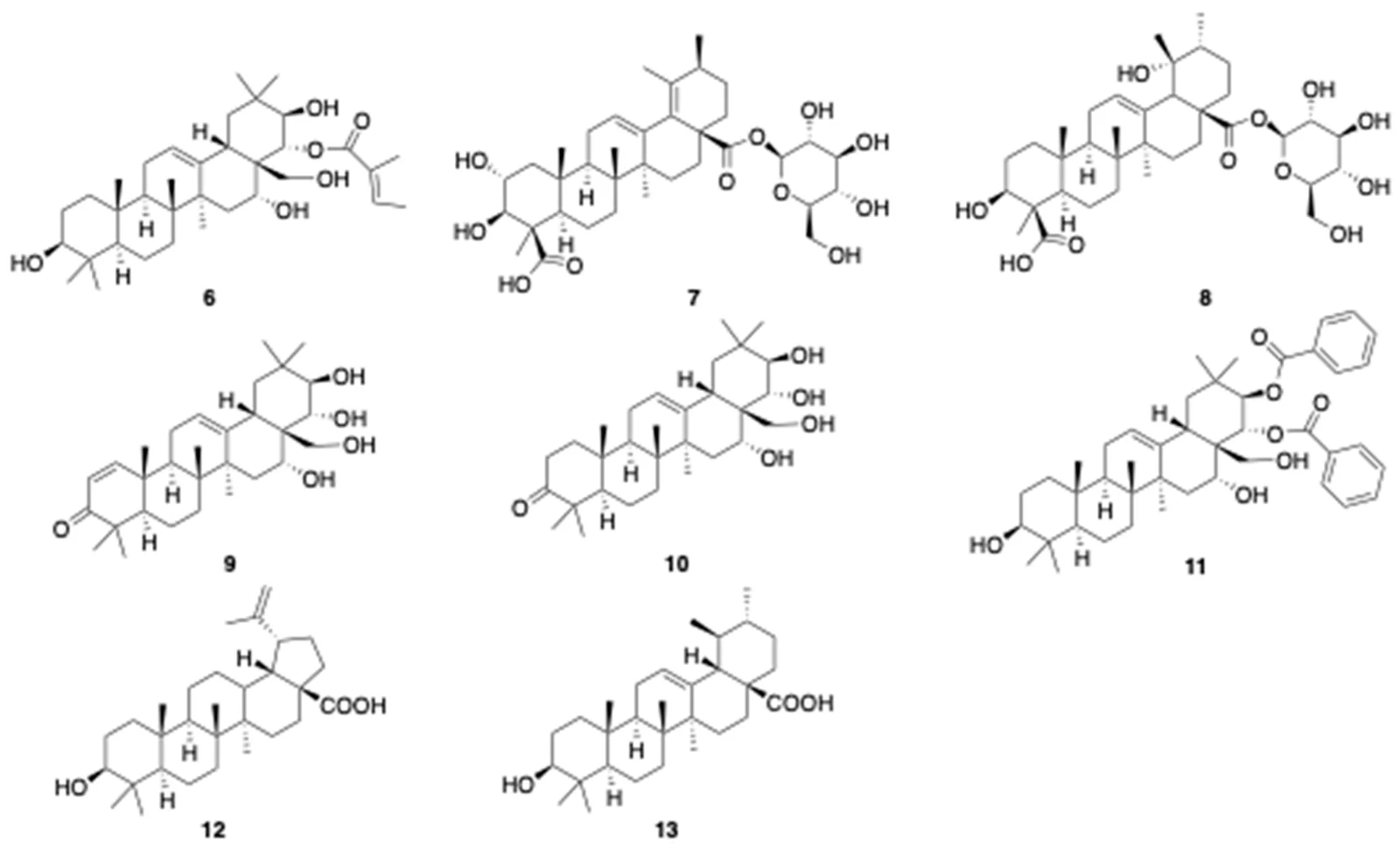
Chemical structures of compounds **6**–**13**.

**Figure 7:**
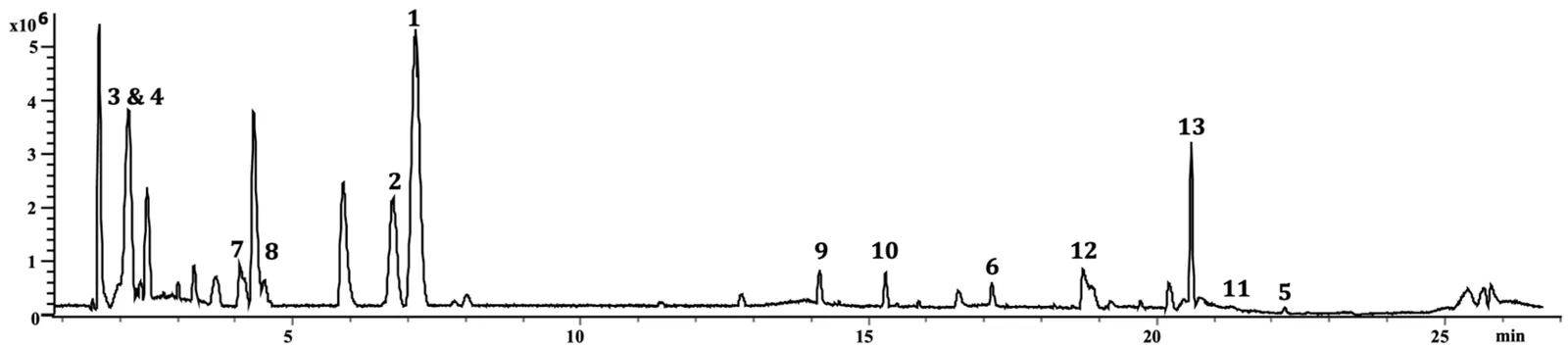
LCMS profile of the aglycone fraction from Mudjala bark. Chromatographic separation was achieved using a Luna C18 reverse-phase analytical column, with an initial 6 min isocratic hold at 40% acetonitrile, followed by a linear gradient to 99% acetonitrile in 20 min.

**Table 1:** NMR data of compound **5** measured at 800 MHz (200 MHz for ^13^C spectra) in DMSO-*d*_6_.

Pos.	δ_C_, Type	δ_H_, Mult (*J* in Hz)	COSY	HMBC	ROESY
1	38.8, CH_2_	1.54, q (3.5)	H-1b, H-2	C-3	
		0.93, d (3.5)	H-1a		
2	27.6, CH_2_	1.52–1.63	H-3	C-3	
3	77.1, CH	3.01, dd (11.1, 5.0)	H-2		3-OH, H-5, H-23
4	38.5, C				
5	55.5, CH	0.69, m	H-6b	C-3, C-10, C-23	H-3, H-27
6	18.6, CH_2_	1.51, m	H-5, H-6b	C-8	
		1.34, m	H-6a, H-7a	C-4	
7	32.7, CH_2_	1.51, m	H-6a	C-26	
		1.28, m		C-5, C-8	
8	40.1, C				
9	46.9, CH	1.60, m	H-11	C-26	
10	38.7, C				
11	23.7, CH_2_	1.85, d (8.9, 3.7)	H-9, H-12	C-8, C-9, C-12, C-13	H-7a, H-12, H-26, H-27
12	123.4, CH	5.32, t (3.7)	H-11	C-9, C-14	H-11, H-18
13	142.3, C				
14	41.8, C				
15	33.7, CH_2_	1.63, m	H-15b, H-16	C-27	H-15b
		1.26, m	H-15a	C-8, C-13, C-16, C-17, C-27	
16	68.1, CH	3.98, dd (6.7, 3.6)	16-OH		16-OH, H-15a, H-15b, H-28b
17	40.1, C				
18	39.5, CH	2.60, m	H-19a	C-12, C-16	H-12, H-21, H-22, H-30
19	46.8, CH_2_	2.60, m	H-18, H-19b	C-13, C-17, C-29	H-19b, H-27
		1.15, m	H-19a	C-13, C-17, C-21	H-19a, H-30
20	36.1, C				
21	79.4, CH	6.01, d (10.2)	H-22	C-20, C-22, C-29, C-30, C-1′	H-22, H-30
22	73.0, CH	5.64, d (10.2)	H-21	C-1″, C-16, C-17, C-21, C-28	H-21, H-29
23	28.7, CH_3_	0.91, s		C-3, C-4, C-5, C-24	H-3, 3-OH, H-6a
24	16.6, CH_3_	0.69, s		C-3, C-5, C-23	H-23, 3-OH
25	17.0, CH_3_	0.85, s		C-10	
26	16.0, CH_3_	0.88, s		C-9, C-15	
27	27.6, CH_3_	1.44, s		C-8, C-13, C-15	H-11, 16-OH, H-19a
28	63.0, CH_2_	2.82, d (10.2)	H-28a	C-18, C-22	H-15a, 28b
		3.07, d (10.2)	H-28b	C-22	H-16, H-28a
29	20.2, CH_3_	1.07, s		C-19, C-20, C-21, C-30	H-19a, H-22
30	29.6, CH_3_	0.82, s		C-19, C-20, C-21, C-29	
1′	166.1, C				
2′	130.8, C				
3′	129.1, CH	7.85, dd (8.2, 1.2)	H-3″	C-1′, C-5′, C-7′	H-4″
4′	128.4, CH	7.49, dd (8.2, 7.4)	H-2″	C-2′, C-6′	H-3″
5′	133.0, CH	7.61, dddd (8.2, 7.4, 1.2, 1.2)		C-3′, C-7′	
6′	128.4, CH	7.49, dd (8.2, 7.4)	H-6″	C-2′, C-4′	H-7″
7′	129.1, CH	7.85, dd (8.2, 1.2)	H-5″	C-1′, C-3′, C-5′	H-6″
1″	167.4, C				
2″	129.3, C				
3″	136.5, CH	6.60, qq (7.2, 1.2)	H-4′	C-1″, C-4″	
4″	14.4, CH_3_	1.52, q (1.2)		C-1″, C-2″, C-3″, C-5″	H-15b
5″	12.5, CH_3_	1.60, dq (7.2, 1.2)	H-3′	C-2″, C-3″, C-4″	H-18, H-19, H-4″
28-OH		4.46, br s	H-3		H-22, H-28a, H-28b
16-OH		4.89, d (3.8)	H-16	C-17	H-15b, H-16, H-21, H-27
3-OH		4.31 br s			H-1b, H-2, H-3

**Table 2:** List of compounds identified from two geographical varieties of *B. acutangula*.

Compound Name	Presence in *B. acutangula* (Indian Variety)	Presence in Mudjala
Bartogenic acid	YES	YES
Barrigenic acid	YES	NO
Barringtogenol B	YES	NO
Barringtogenol C	YES	NO
Barringtogenol E	YES	YES
Barrinic acid	YES	NO
Acutangulic acid	YES	NO
28-O-β-D-glucopyranosylbarrinic acid	YES	NO
Taraxarol	YES	NO
Tormentic acid	YES	NO
Tanginol	YES	NO
Ilexsaponin A1	YES	YES
Ilexgenin A	YES	NO
Trachelosperogenin A	NO	YES
28-O-β-D-glucopyranosylbartogenic acid	NO	YES
Trachelosperoside A1	NO	YES
Chakasapogenin II	NO	YES
Chestnoside B	NO	YES
Cyrrillin A	NO	YES
Bemeuxin	NO	YES
Ursolic acid	NO	YES
Betulinic acid	NO	YES
21-O-benzoyl-22-O-tigloylbarringtogenol C	NO	YES

## Data Availability

The raw data supporting the conclusions of this article will be made available by the authors upon request.

## Supplementary Materials

The following supporting information can be downloaded at https://www.mdpi.com/article/10.3390/molecules31193388/s1, Figure S1: ^1^H-NMR spectrum of previously reported bartogenic acid in DMSO-d6; Figure S2: ^1^H-NMR spectrum of Compound **5** (800 MHz in DMSO-d_6_); Figure S3: ^1^H-^1^H COSY spectrum of Compound **5** (800 MHz in DMSO-d_6_); Figure S4: HSQC spectrum of Compound **5** (800 MHz in DMSO-d_6_); Figure S5: HMBC spectrum of Compound **5** (800 MHz in DMSO-d_6_); Figure S6: ROESY spectrum of Compound **5** (800 MHz in DMSO-d_6_); Figure S7: HRESIMS of Compound **5**; Table S1: Comparison of the NMR spectroscopic data of 28-O-β-D-glucopyranosyl bartogenic acid (**3**) with previously reported data; Table S2: NMR data of bemeuxin (**10**) measured at 800 MHz in DMSO-d_6_; Table S3: ^1^H NMR data of Compounds **1**, **2**, **4**, **6**–**13** measured at 800 MHz in DMSO-d_6_; Table S4: ^13^C NMR data (from HSQC and HMBC) of Compounds **1**, **2**, **4**, **6**–**13** measured at 800 MHz in DMSO-d_6_; Table S5: Summary of characterised compounds from Mudjala bark extract; Table S6: NMR data comparison among Compounds **5**, **6** and the reported data for Compound **6**. References [21,23,26,28,29,30,31,32,33,35] are cited in the Supplementary Materials.
