# Supplementary material for: Discovery of a New Triterpenoid and Chemical Profiling of Mudjala (Barringtonia acutangula), an Australian Indigenous Medicinal Plant
Source: Molecules. 2026 Sep 23;31(19):3388. doi: 10.3390/molecules31193388 (PMC13665909; doi:10.3390/molecules31193388)
Supplement: Supplementary file 1 [file molecules-31-03388-s001.zip › molecules-4546001-supplementary.pdf]

Supplementary Material

for

Discovery of a New Triterpenoid and Chemical Profiling of Mudjala (*Barringtonia acutangula*), an Australian Indigenous Medicinal Plant

Tabassum Jannat [1], Jianying Han [2], Matilda Houston [1], Tin Mak [1], John Watson [3], Anthony Watson [3], Paul W. Marshall [4], Miaomiao Liu [1,*] and Ronald J. Quinn [1,*]

[1] Institute for Biomedicine and Glycomics, Griffith University, Gold Coast, QLD 4215, Australia; tabassum.jannat@griffithuni.edu.au (T.J.); matilda.houston@griffithuni.edu.au (M.H.); t.mak@griffith.edu.au (T.M.)
[2] Institute for Molecular Bioscience, The University of Queensland, Brisbane, QLD 4072, Australia; jianying.han@uq.edu.au
[3] Kimberley Land Council, 11 Gregory St, Broome, WA 6725, Australia; john.watson@klc.org.au (J.W.); anthony.watson@klc.org.au (A.W.)
[4] School of Regulation and Global Governance (RegNet), College of Law, Policy and Governance, Australian National University, Canberra, ACT 2600, Australia; paul.marshall1@anu.edu.au
* Correspondence: miaomiao.liu@griffith.edu.au (M.L.); r.quinn@griffith.edu.au (R.J.Q.)

Table of Contents

Table S1: Comparison of the NMR spectroscopic data of 28-O-β-D-glucopyranosyl bartogenic acid (**3**) with previously reported data 3
Table S2: NMR data of bemeuxin (**10**) measured at 800 MHz in DMSO-d_6........ 4
Table S3: ^{1}H NMR data of Compounds **1**, **2**, **4**, **6**–**13** measured at 800 MHz in DMSO-d_6 5
Table S4: ^{13}C NMR data (from HSQC and HMBC) of Compounds **1**, **2**, **4**, **6**–**13** measured at 800 MHz in DMSO-d_6........ 8
Table S5: Summary of characterised compounds from Mudjala bark extract........ 9
Table S6: NMR data comparison among Compounds **5**, **6** and the reported data for Compound **6** ... 10
Figure S1: ^{1}H-NMR spectrum of previously reported bartogenic acid in DMSO-d_6........ 12
Figure S2: ^{1}H-NMR spectrum of Compound **5** (800 MHz in DMSO-d_6) 12
Figure S3: ^{1}H-^{1}H COSY spectrum of Compound **5** (800 MHz in DMSO-d_6)........ 13
Figure S4: HSQC spectrum of Compound **5** (800 MHz in DMSO-d_6) 13
Figure S5: HMBC spectrum of Compound **5** (800 MHz in DMSO-d_6)........ 14
Figure S6: ROESY spectrum of Compound **5** (800 MHz in DMSO-d_6) 14
Figure S7: HRESIMS of Compound **5** 15
References: 16

Table S1: Comparison of the NMR spectroscopic data of 28-O-β-D-glucopyranosyl bartogenic acid (**3**) with previously reported data

Position	δ_C,[a]	δ_C,[b] (literature)	Difference	δ_H,[a] *mult* (*J* in Hz)	δ_H,[b] *mult* (*J* in Hz)	Difference
**1**	47.2	48.0	-0.8	0.76 td (8.8, 4.6)	0.91 dd (12.0, 11.2)	-0.15
				1.82 dd (8.8, 4.6)	2.01 m	-0.19
**2**	68.1	68.6	-0.5	3.92 td (9.5, 4.6)	4.09 td (11.2, 9.7, 4.7)	-0.17
**3**	82.9	84.3	-1.4	2.7 d (9.5)	2.88 d (9.7)	-0.18
**4**	49.4	50.1	-0.07			
**5**	56.6	57.0	-0.4	0.91 m	1.05 dd (10.4, 4.1)	-0.14
**6**	20.6	20.8	-0.2	1.67 br s	1.81 m	-0.14
**7**	32.8	33.0	-0.2	1.23 m	1.47 ddd (12.9, 12.3, 6.0)	-0.24
				1.36 dd (12.8, 7.4)	1.36 ddd (12.9, 3.4, 3.4)	
**8**	39.6	40.2	-0.06			
**9**	46.6	47.9	-1.3	1.6 m	1.75 m	-0.15
**10**	39.0	39.1	-0.01			
**11**	23.5	24.4	-0.9	1.87 dd (9.9, 3.8)	2.00 m	-0.13
**12**	122.4	124.0	-1.6	5.25 overlapped	5.34 t (3.7)	-0.09
**13**	143.8	144.3	-0.05			
**14**	41.6	42.2	-0.06			
**15**	36.6	28.9	7.7	1.64 m	1.68 m	-0.04
				1.54 m	1.03 m	0.51
**16**	25.1	28.0	-2.9	1.61 m	2.32 ddd (13.1, 12.8, 3.6)	-0.71
					1.73 m	
**17**	45.8	46.5	-0.07			
**18**	53.0	44.7	8.3	2.92 br s	3.06 br s	-0.14
**19**	77.4	80.9	-3.5	3.14 m	3.27 d (3.9)	-0.13
**20**	35.3	35.7	-0.04			
**21**	27.0	29.1	-2.1	1.63 m	1.76 m	-0.13
				0.87 m	1.01 m	-0.14
**22**	31.8	33.4	-1.6	2.23 td (12.8, 3.7)	1.67 m	0.56
					1.78 m	
**23**	25.4	25.2	0.2	1.26 s	1.43 s	-0.17
**24**		180.1	-180.1			
**25**	15.1	17.5	-2.4	0.84 s	0.95 s	-0.11
**26**	16.4	15.2	1.2	0.64 s	0.77 s	-0.13
**27**	24.1	24.8	-0.7	1.23 s	1.3 s	-0.07
**28**	178.6	177.3	1.3			

**29**	28	28.8	-0.8	0.85 s	0.94 s	-0.09
**30**	24.4	24.6	-0.2	0.87 s	0.95 s	-0.08
**Gluc.**						
**1'**	93.8	95.9	-2.1	5.24 m	5.38 d (8.1)	-0.13
**2'**	72.3	74.1	-1.8	3.2 m	3.32 dd (8.1, 9.5)	-0.12
**3'**	80	79.0	1.0	3.15 m	3.41 m	-0.26
**4'**	69.4	71.0	-1.6	3.10 d (10.6)	3.36 m	-0.26
**5'**	76.5	79.4	-2.9	3.10 d (10.6)	3.35 m	-0.25
**6'**	60.4	62.1	-1.7	3.6 dt (11.9, 5.4)	3.82 dd (12.0, 1.6)	-0.22
				3.45 dt (11.9, 2.7)	3.68 dd (12.0, 4.5)	-0.23

[a] Obtained through HMBC and HSQC, measured at 800 MHz in DMSO-d_6
[b] Literature data measured at 400 MHz (100 MHz for ^{13}C spectra) in $CDCl_3$ [1]

Table S2: NMR data of bemeuxin (**10**) measured at 800 MHz in DMSO-d_6

Position	δ_C	δ_H, *mult* (*J* in Hz)	COSY	HMBC
**1**	39.2	1.80, ddd (13.1, 7.6, 3.9)	H-1b, H-2a	C-5
		1.42, ddd (J = 14.7, 7.8, 3.1)	H-1a	C-5, C-25
**2**	34.0	2.50, m	-	C-4
		2.32, m	-	C-4
**3**	204.3			
**4**	47.2			
**5**	54.8	1.33, dd (J = 7.0, 3.6)	H-6	C-7, C-24, C-25
**6**	19.0	1.44, dd (J = 5.3, 3.6)	H-5	C-8
**7**	31.8	1.53, td (J = 7.4, 4.9)	H-7b	C-5, C-9
		1.31, dd (J = 7.8, 3.4)	H-7a	C-5, C-9
**8**	39.6			
**9**	45.9	1.67, dd (J = 6.3, 4.7)	H-11a	C-8, C-11, C-14, C-25, C-26
**10**	39.8			
**11**	23.7	1.88, ddd (J = 18.3, 11.5, 3.2)	H-9	C-9, C-12, C-13
		1.84, dq (J = 18.1, 4.2)	H-9	C-9, C-12, C-13
**12**	122.1	5.23, t (3.7)	H-11	C-9, C-14, c-18
**13**	143.6			
**14**	41.6			
**15**	33.7	1.66, dq (J = 15.5, 4.3)	H-15b, H-16	C-27
		1.23, dt (11.2, 2.5)	H-15a	C-9, C-13, C-16, C-27
**16**	67.1	4.04, s	H-15, 16-OH	C-14, c-18
**17**	46.5			
**18**	40.0	2.3, dd (J = 9.6, 3.2)	H-19	C-13, C-14, C-16, C-19
**19**	47.6	2.35, d (J = 12.4)	H-19b	C-18, C-29
		0.94, dd (J = 9.1, 3.3)	H-19a	C-21
**20**	35.9			
**21**	77.3	3.79, d (9.7)	H-22	C-22, C-29, C-30
**22**	74.5	3.61, d (9.7)	H-21	C-16, C-17, C-28
**23**	26.8	1.00 s		C-3, C-4, C-5, C-24

**24**	21.6	0.97, s		C-4, C-5, C-23
**25**	15.3	0.99, s		C-8, C-9
**26**	16.4	0.89, s		C-8, C-9, C-14
**27**	27.3	1.37, s		C-8, C-14, C-15
**28**	65.5	3.17, d (10.2)	H-28b	C-16, C-17, C-18, C-22
		3.00, d (10.2)	H-28a	C-16, C-17, C-18, C-22
**29**	30.5	0.84, s		C-19, C-20, C-21, C-30
**30**	19.2	0.81, s		C-19, C-20, C-21, C-29
**16- OH**		4.31, d (4.4)		

Table S3: ^{1}H NMR data of Compounds **1**, **2**, **4**, **6–13** measured at 800 MHz in DMSO-d_6

Position	1	2	4	6	7	8	9	10	11	12	13
**1**	1.82 dd (8.8, 4.6)	1.78 dd (12.6, 4.5)	1.82 dd (12.0, 4.6)	1.51, dt (6.1, 3.6)	1.95 m	1.63 m	7.18 d (10.2)	1.80, ddd (13.1, 7.6, 3.9)	1.54 m	1.58 dt (13.2, 3.5)	1.57 m
	0.76 td (8.8, 4.6)	0.65 t (12.6)	0.70 d (12.0)	0.92, p (6.1)	0.82, m	0.99 m		1.42, ddd (J = 14.7, 7.8, 3.1)	0.95 m	0.85 dd (12.4, 4.3)	1.52 m
**2**	3.92 td (9.5, 4.6)	2.73 d (9.5)	3.83 br s	1.48, t, J = 3.0 Hz	3.89, br s	1.98 m	5.73 d (10.2)	2.50, m	1.70 m	1.47 m	1.45 m
						1.56 m		2.32, m	1.45 m		
**3**	2.70 d (9.5)	3.78 dd (9.5, 4.5)	2.57 br s	3.00 dd, J = 5.6, 4.9 Hz	2.7, br s	3.04 dd (11.9, 4.5)			3.03 m	3.00 dd (10.9, 5.3)	3.00 m
**4**											
**5**	0.91 m	0.78 m	0.82 m	0.68, d, J = 12.3	0.88 m	0.88 m	1.23 m	1.33, dd (J = 7.0, 3.6)	0.71 m	0.65 d (10.5)	0.68 m
**6**	1.67 br s	1.67 d (12.8)	1.63 m	1.47, d (5.3)	1.66, m	1.69 m	1.57 m	1.44, dd (J = 5.3, 3.6)	1.51 m	1.47 m	1.48 m
		1.97 (3.2)	1.23 m	1.31, dd (5.3, 3.6)		1.64 m	1.38 m		1.34 m	1.34 m	1.31 m
**7**	1.23 m	1.25 d (9.5)	1.81 m	1.25, t (5.3)	1.24 m	1.26 m	1.20 m	1.31, dd (J = 7.8, 3.4)	1.33 m	1.34 m	1.27 m
	1.36 dd (12.8, 7.4)	1.37 m	1.35 m	1.44, dt (5.3, 3.6)		1.38 m	1.51 m	1.53, td (J = 7.8, 3.4)	1.53 m		1.45 m
**8**											
**9**	1.6 m	1.56 m	1.54 m	1.57, dd (9.8, 4.0)	1.32 m	1.55 m	1.26 m	1.67, dd (J = 6.3, 4.7)	1.61 m	1.27 m	1.47 m
**10**											
**11**	1.87 dd (9.9, 3.8)	1.91 m	1.92 m	1.81, dd (8.9, 3.7)	1.85 m	1.93 m	1.82 dd (11.8, 3.6)	1.88, ddd (J = 18.3, 11.5, 3.7)	1.86 m	1.38 m	1.85 m
			1.92 m			1.84 m		1.84, dq (J = 18.3, 3.7)		1.17 m	
**12**	5.25[C]	5.18 t (4.2)	5.18[C]	5.23 t (3.8)	5.27[C]	5.18 t (3.6)	5.28 t (3.6)	5.23, t (3.7)	5.34 t (3.6)	1.64 d (13.2)	5.13 t (3.6)
										0.99 dd (13.2, 4.3)	
**13**										2.24 td (12.7, 3.1)	

**14**											
**15**	1.64 m	1.59 m	1.63 m	1.56, d (2.1)	1.85 m	1.77 m,	1.67 dd (14.9, 4.8)	1.66, dq (J = 15.5, 4.3)	1.66 m	1.41 m	1.80 m
	1.54 m	1.50 td (13.0, 4.1)	1.48 dd (8.3, 2.3)	1.21, dd (14.5, 2.1)	1.07 m	0.89 m	1.25, m	1.23, dt (11.2, 2.5)	1.29 m	1.11 m	1.01 m
**16**	1.61 m	2.47[C]	2.52[C]	3.89, t (2.1)	2.17 td (13.9, 8.3)	2.53 m,	4.03 t (4.8)	4.04, s	4.13 brs	2.13 dt (12.3, 3.5)	1.93 m
		1.37 m	1.51 br s		1.41 m	1.51 m				1.37 m	1.52 m
**17**											
**18**	2.92 br s	2.37 br s	2.38 br s	2.45, dd (11.0, 3.5)		2.38 brs	2.30 m	2.3, dd (J = 9.6, 3.2)	2.63 m	1.54 t (11.4)	2.10 d (11.2)
**19**	3.14 m			2.43, d (11.0)			2.38 m	2.35, d (J = 12.4)	2.65 m	2.96 m	1.31 m
				1.00, td (3.9, 3.5)			0.92 br s	0.94, dd (J = 9.1, 3.3)	1.19 m		
**20**		1.27 m	1.26 d (6.5)		2.13, m	1.26 m					1.31 m
**21**	1.63 m	1.24 d (4.9)	1.63 m	4.06, d (9.8)	1.63 m	1.63 m,	3.80 dd (10.2, 2.4)	3.79, d (9.7)	6.12 d (10.1)	1.83 m	1.44 m
	0.87 m		1.16 dd (8.3, 2.3)		1.29 m	1.16 m	3.62 dd (10.2, 2.4)			1.32 m	1.28 m
**22**	2.23 td (12.8, 3.7)	1.69 m	1.76 m	5.16, d (9.8)	1.79 d (13.4)	1.64 m		3.61, d (9.7)	5.85 d (10.1)	1.81 m	1.54 m
		0.90 m	0.88 m		1.57 m	1.48 m				1.43 m	
**23**	1.26 s	1.21 s	1.20 s	0.90, s	1.26 m	1.28 s	1.07 s	1.00 s	0.92 s	0.89 s	0.68 s
**24**				0.68, s			1.01 s	0.97, s	0.69 s	0.67 s	0.89 s
**25**	0.84 s	0.79 s	0.85 s	0.87, s	0.88 s	0.83 s	1.13 s	0.99, s	0.89 s	0.78 s	0.86 s
**26**	0.64 s	0.73 s	0.66 s	0.82, s	0.82 s	0.69 s	0.85 s	0.89, s	0.85 s	0.88 s	0.75 s
**27**	1.23 s	1.28 s	1.26 s	1.38, s	0.91 s	1.29 s	1.38 s	1.37, s	1.47 s	0.95 s	1.04 s
**28**				2.98, d (10.6)			3.18 d (10.2)	3.17, d (10.2)	3.13 dd (10.7, 4.4)		
				2.74, d (10.6)			3.01 d (10.2)	3.00, d (10.2)	2.92 dd (10.3, 4.2)		
**29**	0.87 s	0.84 d (6.4)	0.84 d (6.5)	0.85, s	1.70 s	0.85 d (6.7)	0.82 s	0.84, s	0.85 s	4.71 s	0.81 d (6.5)
										4.58 s	
**30**	0.85 s	1.08 s	1.09 s	0.84, s	1.04 d (7.1)	1.09 s	0.95 s	0.81, s	1.13 s	1.67 s	0.91 d (5.9)
**28-O-gluc.**											
**1'**			5.18[C]		5.26 q (2.6)	5.16 d (8.2)					
**2'**			3.20 m		3.07, m	3.10 t (8.6)					
**3'**			3.14 d (10.2)		3.17, m	3.19 t (8.8)					
**4'**			3.10 d (10.2)		3.10, m	3.12 m					
**5'**			3.10 d (10.2)		3.20, m	3.13 m					
**6'**			3.60 m		3.60 d (10.8)	3.60 d (11.7),					

	3.45 m		3.45^{C}	3.44 m					
**22-O-tigloyl**									
**1”**									
**2”**									
**3”**		6.81 qq (7.0, 1.2)							
**4”**		1.76 dq (7.0, 1.2)							
**5”**		1.79 q (1.2)							
**21-O-benzoyl**									
**1’**									
**2’**									
**3’**							7.83 dd (8.2, 1.3)		
**4’**							7.43 t (7.9)		
**5’**							7.55 tt (7.4, 1.2)		
**6’**							7.43 t (7.9)		
**7’**							7.83 dd (8.2, 1.3)		
**22-O-benzoyl**									
**1’**									
**2’**									
**3’**							7.80 dd (8.2, 1.3)		
**4’**							7.39 t (7.9)		
**5’**							7.53 tt (7.4, 1.2)		
**6’**							7.39 t (7.9)		
**7’**							7.80 dd (8.2, 1.3)		
**28-OH**		4.59 m					4.57 t (4.3)		
**16-OH**		4.30 br s			4.31, d (4.4)		5.09 d (4.2)		4.28 d (5.2
**21-OH**		4.14 br s							
**3-OH**		4.14 br s					4.30 d (5.1)		
**28-COOH**									11.93 s

C = overlapped by solvent/H_2O/another peak

Table S4: ^{13}C NMR data (from HSQC and HMBC) of Compounds **1**, **2**, **4**, **6–13** measured at 800 MHz in DMSO-d_6

Position	1	2	4	6	7	8	9	10	11	12	13
**1**	47.5	46.9	48.2	38.8	48.5	39.2	159.4	39.2	38.7	38.7	36.8
**2**	67.4	68.1	68.1	27.6	67.4	28.3	125.0	34.0	27.5	27.6	27.5
**3**	82.5	82.3	83.5	77.4	82.9	77.2	204.3	204.3	77.2	77.2	77.3
**4**	49.4	49.4	56.3	27.6	49.1	48.5	44.4	47.2	38.9	39.0	38.7
**5**	55.7	55.3	55.9	55.2	56.2	55.9	48.8	54.8	55.5	55.4	55.2
**6**	20.2	20.2	20.5	18.6	20.6	20.3	18.9	19.0	18.6	18.4	18.5
**7**	33.0	33.1	33.7	32.7	33.4	33.3	32.7	31.8	32.6	34.4	33.2
**8**	39.4	39.4	41.6	39.6	39.2	41.7	39.8	39.6	40.5	40.7	39.9
**9**	47.2	45.6	46.5	46.5	46.9	46.5	46.3	45.9	46.8	50.4	47.5
**10**	38.5	38.5	39.8	37.0	39.0	37.0	40.0	39.8	36.4	37.2	39.0
**11**	23.7	23.5	24.0	23.4	23.7	23.8	23.4	23.7	23.7	20.9	23.3
**12**	123.0	126.6	127.5	122.1	125.9	127.6	122.4	122.1	123.4	25.5	125.0
**13**	143.6	139.3	138.6	143.0	138.4	138.6	143.6	143.6	142.3	38.1	138.7
**14**	41.6	41.6	46.5	41.3	44.6	42.1	41.8	41.6	40.5	42.5	42.1
**15**	32.7	37.3	28.5	34.0	28.5	28.6	33.7	33.7	34.2	29.7	28.0
**16**	27.3	25.1	25.7	68.1	35.0	25.6	67.1	67.1	67.7	32.2	24.3
**17**	45.2	45.2	47.8	46.8	53.7	47.9	46.5	46.5	47.2	55.9	47.3
**18**	43.7	53.3	53.9	39.8	133.2	53.7	39.8	40.0	39.8	49.0	52.8
**19**	80.6	72.1	72.1	47.2	135.6	72.1	47.8	47.6	46.9	47.1	38.9
**20**	35.3	41.5	41.7	36.4	34.0	41.7	35.3	35.9	36.2	150.8	38.9
**21**	28.6	28.9	26.3	75.1	26.3	26.3	77.1	77.3	79.5	30.6	30.6
**22**	27.6	28.0	37.2	76.1	30.5	37.4	74.5	74.5	74.0	36.8	37.0
**23**	24.7	24.7	25.7	28.9	25.0	24.3	27.6	26.8	28.8	28.6	16.6
**24**	178.5	176.1	179.6	16.7	178.9	178.7	21.8	21.6	16.7	16.3	28.7
**25**	14.7	15.4	24.0	16.0	16.0	13.8	18.9	15.3	15.6	16.4	15.7
**26**	17.2	16.7	16.9	17.0	18.3	16.9	17.0	16.4	17.0	16.2	17.4
**27**	24.4	23.8	24.4	27.3	22.1	24.2	27.3	27.3	27.7	14.8	23.7
**28**	180	179.6	176.1	63.5	172.6	176.1	65.5	65.5	63.5	177.7	178.7
**29**	25.1	16.4	16.7	19.2	19.5	16.7	19.5	19.2	20.2	110.1	17.5
**30**	28.5	26.3	26.9	30.2	18.9	26.9	30.2	30.5	29.6	19.4	21.5
**28-O-gluc.**											
**1'**			94.4		94.4	94.6					
**2'**			77.1		72.9	72.7					
**3'**			78.0		78.0	77.2					
**4'**			70.0		70.0	70.0					
**5'**			84.5		77.1	78.1					
**6'**			61.0		61.3	61.1					

**22-O-tigloyl**		
**1”**	169.0	
**2”**	129.6	
**3”**	136.5	
**4”**	14.5	
**5”**	12.8	
**21-O-benzoyl**		
**1’**		165.7
**2’**		130.2
**3’**		129.1
**4’**		128.5
**5’**		133.0
**6’**		128.5
**7’**		129.1
**22-O-benzoyl**		
**1’**		165.7
**2’**		130.2
**3’**		129.1
**4’**		128.5
**5’**		133.0
**6’**		128.5
**7’**		129.1

Table S5: Summary of characterised compounds from Mudjala bark extract

Compound No.	Trivial Name	Molecular formula	Isolation amount	Identification method	Reference
**1**	Bartogenic acid	$C_{30}H_{46}O_7$	0.5 mg (from hydrolysis)	1D and 2D NMR, HRESIMS	[2]
**2**	Trachelosperogenin A	$C_{30}H_{46}O_7$	0.2 mg (from hydrolysis)		[2]
**3**	28-O-β-D-Glucopyranosylbartogenic acid	$C_{36}H_{56}O_{12}$	10.0 mg		[1]
**4**	Trachelosperoside A1	$C_{36}H_{56}O_{12}$	4.0 mg		[2]
**5**	21-O-benzoyl-22-O-tigloylbarringtogenol C (New)	$C_{42}H_{60}O_7$	1.1 mg		
**6**	Chakasapogenin II	$C_{35}H_{56}O_6$	2.8 mg		[3]
**7**	Chestnoside B	$C_{36}H_{54}O_{11}$	0.5 mg		[4]
**8**	Ilexsaponin A1	$C_{35}H_{56}O_6$	0.65 mg		[5]
**9**	Cyrillin A	$C_{30}H_{46}O_5$	1.3 mg		[6]
**10**	Bemeuxin	$C_{30}H_{48}O_5$	3.7 mg		[7]
**11**	Barringtogenol E	$C_{44}H_{58}O_7$	1.0 mg		[8]

**12**	Betulinic acid	$C_{30}H_{48}O_3$	11.0 mg	[9]
**13**	Ursolic acid	$C_{30}H_{48}O_3$	0.93 mg	[10]

Table S6: NMR data comparison among Compounds **5**, **6** and the reported data for Compound **6**

**Position**	**5**[a]		**6**[a]		**Literature value**[b] **[3]**	
	**δc**	**δH, mult (J in Hz)**	**δc**	**δH, mult (J in Hz)**	**δc**	**δH, mult (J in Hz)**
**1**	38.8	1.54, q (3.5)	38.8	1.51, dt (6.1, 3.6)	39.2	
		0.93, d (3.5)		0.92, p (6.1)		
**2**	27.6	1.52-1.63	27.6	1.48, t, (3.0)	28.8	
**3**	77.1	3.01, dd (11.1, 5.0)	77.4	3.00 dd, (5.6, 4.9)	78.1	3.50, m
**4**	38.5		27.6		39.5	
**5**	55.5	0.69, m	55.2	0.68, d, (12.3)	55.8	
**6**	18.6	1.51, m	18.6	1.47, d (5.3)	18.8	
		1.34, m		1.31, dd (5.3, 3.6)		
**7**	32.7	1.51, m	32.7	1.25, t (5.3)	33.3	
		1.28, m		1.44, dt (5.3, 3.6)		
**8**	40.1		39.6		40.1	
**9**	46.9	1.60, m	46.5	1.57, dd (9.8, 4.0)	47.2	
**10**	38.7		37.0		37.3	
**11**	23.7	1.85, d (8.9, 3.7)	23.4	1.81, dd (8.9, 3.7)	24.0	
**12**	123.4	5.32, t (3.7)	122.1	5.23 t (3.8)	123.8	5.52, t
**13**	142.3		143.0		143.3	
**14**	41.8		41.3		41.8	
**15**	33.7	1.63, m	34.0	1.56, d (2.1)	34.9	1.65, dd
		1.26, m		1.21, dd (14.5, 2.1)		1.92, dd
**16**	68.1	3.98, dd (6.7, 3.6)	68.1	3.89, t (2.1)	69.2	4.52, s
**17**	40.1		46.8		48.3	
**18**	39.5	2.60, m	39.8	2.45, dd (11.0, 3.5)	40.5	
**19**	46.8	2.60, m	47.2	2.43, d (11.0)	49.7	
		1.15, m		1.00, td, (3.9, 3.5)		
**20**	36.1		36.4		37.1	
**21**	79.4	6.01, d (10.2)	75.1	4.06, d (9.8)	76.7	5.13
**22**	73.0	5.64, d (10.2)	76.1	5.16, d (9.8)	77.6	6.23, d (10.3)
**23**	28.7	0.91, s	28.9	0.9, s	28.2	1.26, s
**24**	16.6	0.69, s	16.7	0.68, s	16.6	1.08, s
**25**	17.0	0.85, s	16.0	0.87, s	15.9	0.96, s

**26**	16.0	0.88, s	17.0	0.82, s	17.0	0.96, s
**27**	27.6	1.44, s	27.3	1.38, s	27.6	1.87, s
**28**	63.0	2.82, d (10.2)	63.5	2.98, d (10.6)	64.2	3.41, d
		3.07, d (10.2)		2.74, d (10.6)		3.69, d
**29**	20.2	1.07, s	19.2	0.85, s	19.5	1.36, s
**30**	29.6	0.82, s	30.2	0.84, s	30.4	1.46, s
**21-O-benzoyl**						
**1’**	166.1					
**2’**	130.8					
**3’**	129.1	7.85, dd (8.2, 1.2)				
**4’**	128.4	7.49, dd (8.2, 7.4)				
**5’**	133.0	7.61, dddd (8.2, 7.4, 1.2, 1.2)				
**6’**	128.4	7.49, dd (8.2, 7.4)				
**7’**	129.1	7.85, dd (8.2, 1.2)				
**22-O-tigloyl**						
**1”**	167.4		169.0		169.5	
**2”**	129.3		129.6		129.7	
**3”**	136.5	6.60, qq (7.2, 1.2)	136.5	6.81 qq (7.0, 1.2)	136.8	7.04, q (7.0)
**4”**	14.4	1.52, q (1.2)	14.5	1.76 dq (7.0, 1.2)	14.1	1.49, d (7.0)
**5”**	12.5	1.60, dq (7.2, 1.2)	12.8	1.79 q (1.2)	12.4	1.85, s
**28-OH**		4.46, br s		4.59 m		
**16-OH**		4.89, d (3.8)		4.30 br s		
**21-OH**				4.14 br s		
**3-OH**		4.31 br s		4.14 br s		

a = in DMSO-d_6; b = in pyridine-d_5

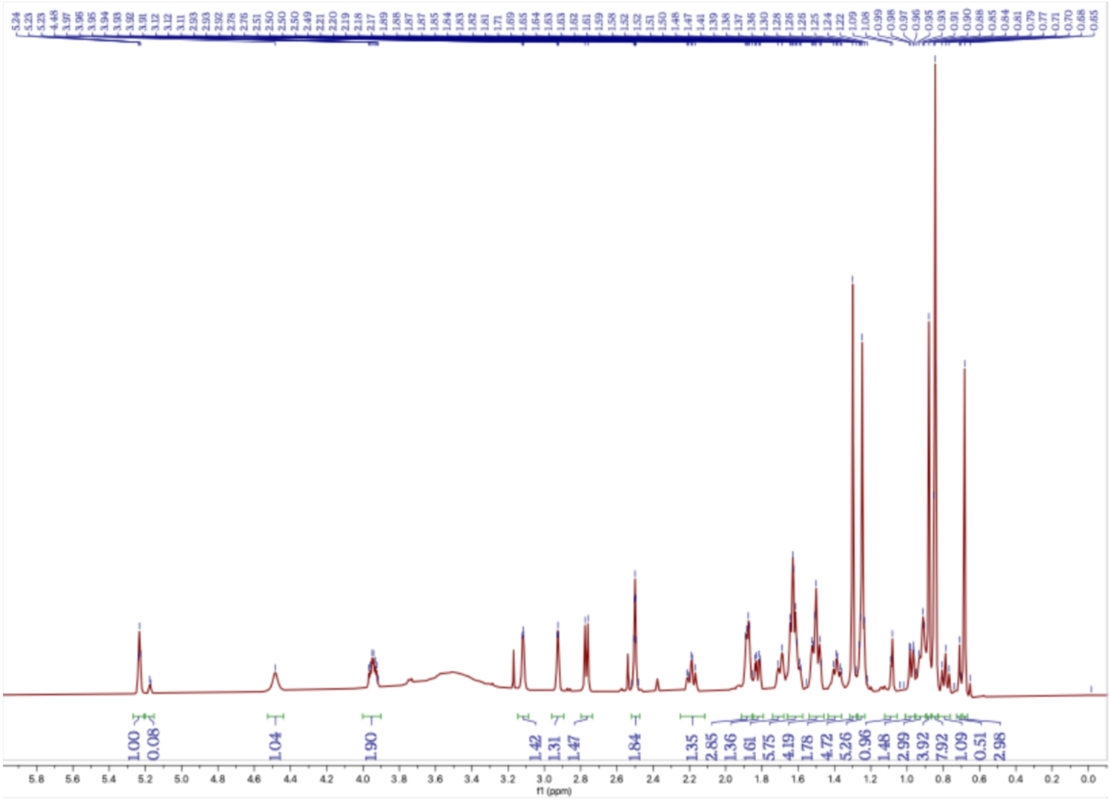

Figure S1: ^{1}H-NMR spectrum of previously reported bartogenic acid in DMSO-d_6

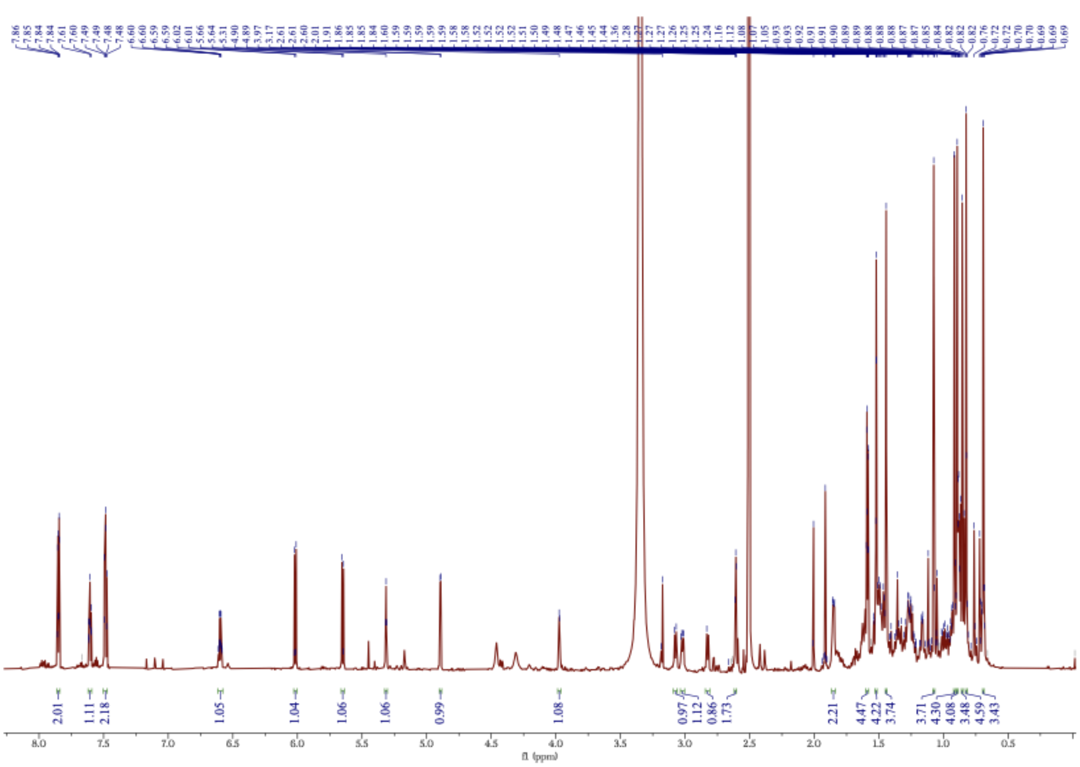

Figure S2: ^{1}H-NMR spectrum of Compound **5** (800 MHz in DMSO-d_6)

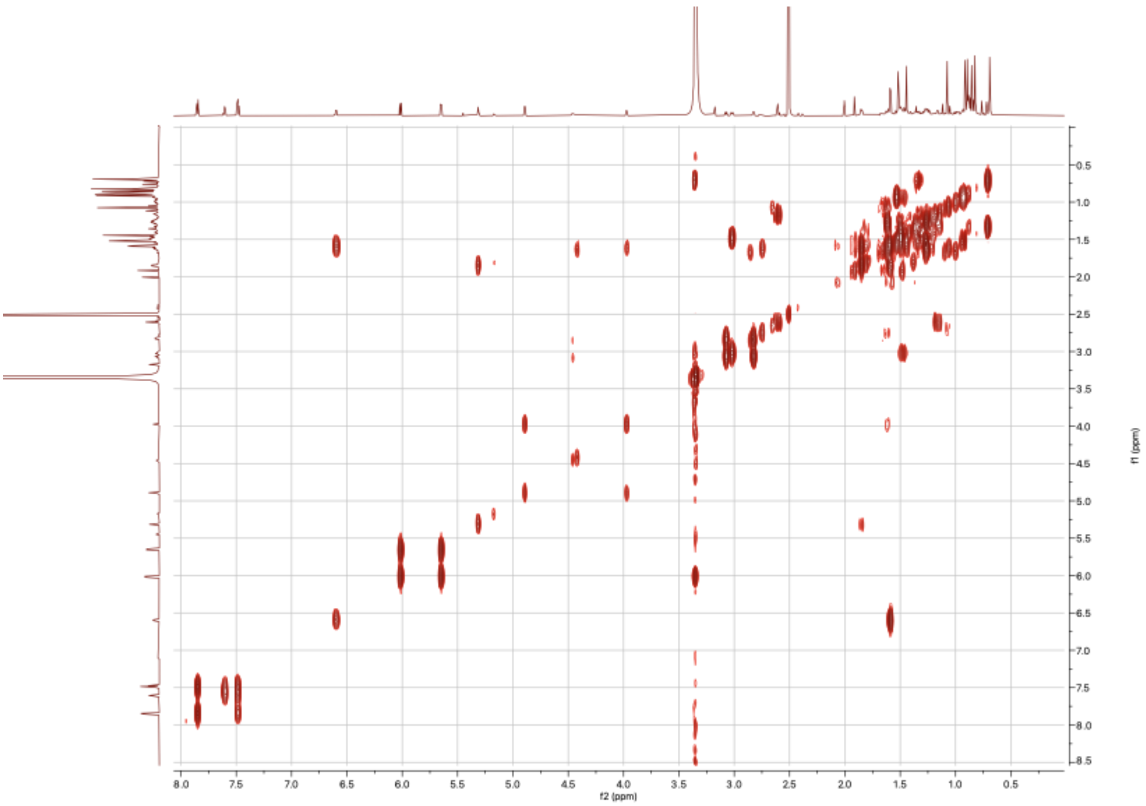

Figure S3: ^{1}H-^{1}H COSY spectrum of Compound **5** (800 MHz in DMSO-d_6)

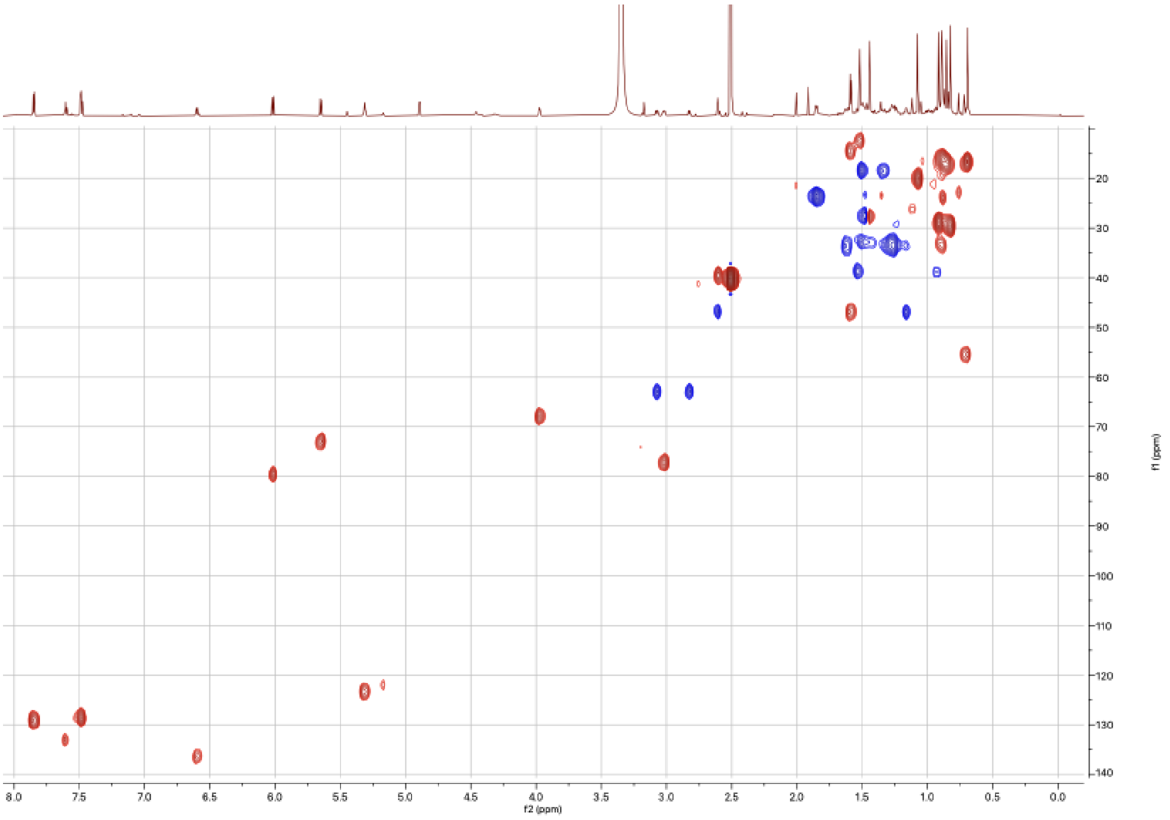

Figure S4: HSQC spectrum of Compound **5** (800 MHz in DMSO-d_6)

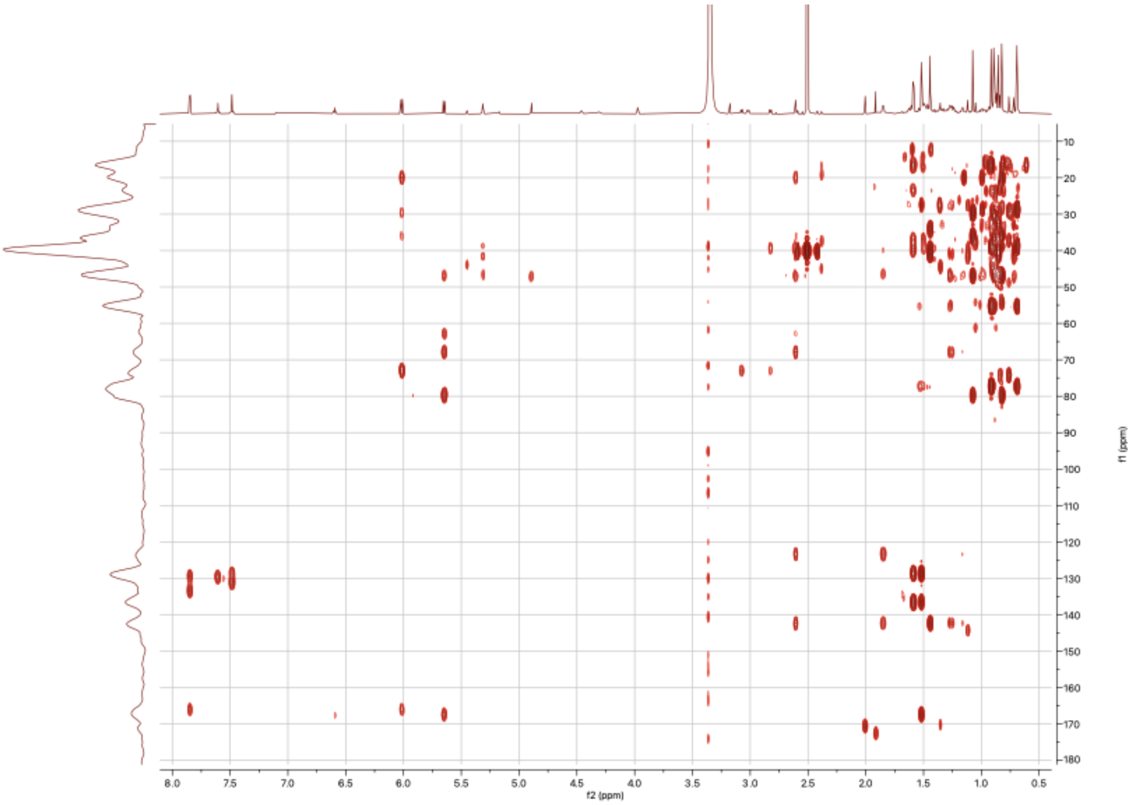

Figure S5: HMBC spectrum of Compound **5** (800 MHz in DMSO-d_6)

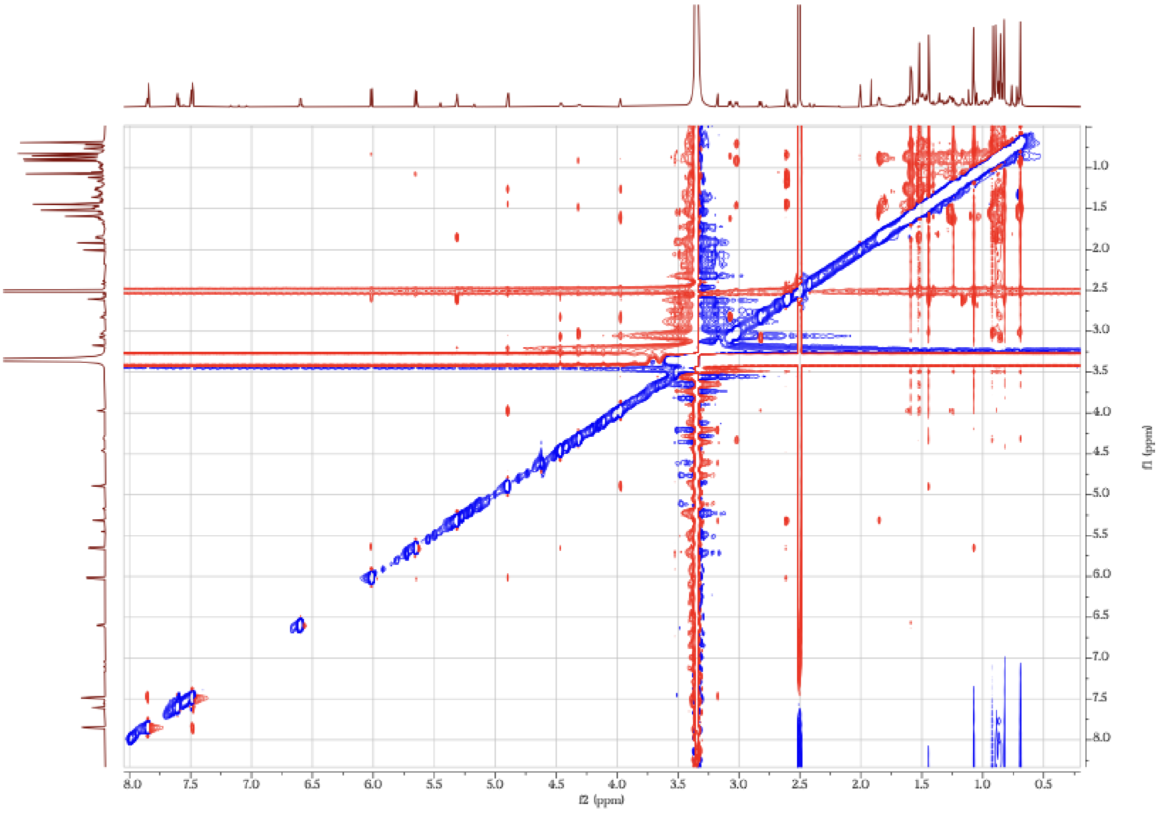

Figure S6: ROESY spectrum of Compound **5** (800 MHz in DMSO-d_6)

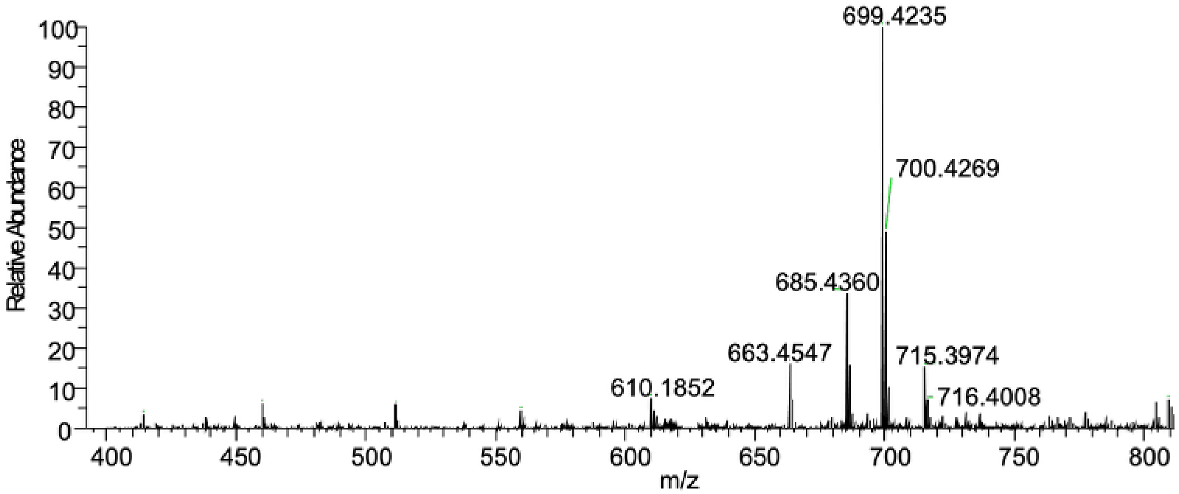

Figure S7: HRESIMS of Compound **5**

References:

1. Arramon, G.; Saucier, C.; Colombani, D.; Glories, Y., Identification of triterpene saponins in Quercus robur L. and Q. petraea Liebl. heartwood by LC-ESI/MS and NMR. *Phytochem. Anal.* **2002,** 13, (6), 305-310.
2. Abe, F.; Yamauchi, T., Trachelosperosides, glycosides of 19-alpha-hydroxyursane-type Triterpenoids from *Trachelospermum asiaticum* (Trachelospermum. III). *Chem. Pharm. Bull.* **1987,** 35, (5), 1748-1754.
3. Yoneda, T.; Nakamura, S.; Ogawa, K.; Matsumoto, T.; Nakashima, S.; Matsumura, K.; Tanaka, A.; Ryu, K.; Fukaya, M.; Fujimuro, M., Oleanane-type triterpenes with highly-substituted oxygen functional groups from the flower buds of Camellia sinensis and their inhibitory effects against no production and HSV-1. *Nat. Prod. Commun.* **2018,** 13, (2), 1934578X1801300206.
4. Pérez, A. J.; Pecio, Ł.; Kowalczyk, M.; Kontek, R.; Gajek, G.; Stopinsek, L.; Mirt, I.; Oleszek, W.; Stochmal, A., Triterpenoid components from oak heartwood (Quercus robur) and their potential health benefits. *J. Agric. Food Chem.* **2017,** 65, (23), 4611-4623.
5. Hidaka, K.; Ito, M.; Matsuda, Y.; Kohda, H.; Yamasaki, K.; Yamahara, J., A triterpene and saponin from roots of *Ilex pubescens*. *Phytochemistry* **1987,** 26, (7), 2023-2027.
6. Ren, Y. L.; VanSchoiack, A.; Chai, H. B.; Goetz, M.; Kinghorn, A. D., Cytotoxic barrigenol-like triterpenoids from an extract of housed in a repository. *J. Nat. Prod.* **2015,** 78, (10), 2440-2446.
7. Wang, M. K.; Wu, F.; Chen, Y., Studies on the triterpenes from *Berneuxia thibetica* Decne. *Yao Xue Xue Bao* **1993,** 28, (11), 845-848.
8. Ma, S. Y.; Weng, M. L.; Yang, T.; Ge, L.; Yang, K. D., Triterpenes and pheophorbides from *Camellia ptilosperma* and their cytotoxicity, photocytotoxicity, and photodynamic antibacterial activity. *Molecules* **2023,** 28, (20).
9. Dais, P.; Plessel, R.; Williamson, K.; Hatzakis, E., Complete ^{1}H and ^{13}C NMR assignment and ^{3}1P NMR determination of pentacyclic triterpenic acids. *Anal. Methods* **2017,** 9, (6), 949-957.
10. Seebacher, W.; Simic, N.; Weis, R.; Saf, R.; Kunert, O., Complete assignments of ^{1}H and ^{13}C NMR resonances of oleanolic acid, 18α-oleanolic acid, ursolic acid and their 11-oxo derivatives. *Magn. Reson. Chem.* **2003,** 41, (8), 636-638.
